# Serum soluble CD26/DPP4 titer variation is a potential prognostic biomarker in cancer therapy with a humanized anti-CD26 antibody

**DOI:** 10.1186/s40364-021-00273-0

**Published:** 2021-03-23

**Authors:** Yutaro Kaneko, Ryo Hatano, Naoto Hirota, Nicolas Isambert, Véronique Trillet-Lenoir, Benoit You, Jérôme Alexandre, Gérard Zalcman, Fanny Valleix, Thomas Podoll, Yoshimi Umezawa, Seiichi Takao, Satoshi Iwata, Osamu Hosono, Tetsuo Taguchi, Taketo Yamada, Nam H. Dang, Kei Ohnuma, Eric Angevin, Chikao Morimoto

**Affiliations:** 1 Y’s AC Co., Ltd., 2-6-8, Kudan-minami, Chiyoda-ku, Tokyo, 102-0074 Japan; 2 Department of Therapy Development and Innovation for Immune Disorders and Cancers, Graduate School of Medicine, Juntendo University, Tokyo, Japan; 3 Stella Co., Ltd., Tokyo, Japan; 4 Centre Georges-François Leclerc, Unité de Phases Précoces, Dijon, France; 5 Institut de Cancérologie des Hospices Civils de Lyon, CITOHL, Lyon, France; 6 Hôpital Cochin, Paris, France; 7 Centre Hospitalier Universitaire (CHU) de Caen, Centre de Recherche Clinique/Essais de phases précoces, Caen, France; 8 FV Clinical subcontractor for SynteractHCR SAS, Levallois-Perret, France; 9 Y’s therapeutics Inc., Redwood City, CA USA; 10 Department of Rheumatology and Allergy, IMSUT Hospital, The University of Tokyo, Tokyo, Japan; 11 Graduate School of Medicine, Osaka University, Osaka, Japan; 12 Saitama Medical University, Saitama, Japan; 13 Keio University School of Medicine, Tokyo, Japan; 14 Division of Hematology/Oncology, University of Florida, Gainesville, FL USA; 15 Gustave Roussy, Drug Development Department (DITEP), Université Paris-Saclay, Villejuif, France

**Keywords:** Serum soluble CD26/dipeptidyl peptidase 4, YS110, Prognostic biomarker, Cancer therapy

## Abstract

**Background:**

The phase I trial of the humanized anti-CD26 monoclonal antibody YS110 for CD26-expressing tumors was conducted recently. The present study identifies a potential prognostic biomarker for CD26-targeted therapy based on the phase I data.

**Methods:**

*Box and Whisker plot* analysis, *Scatter plot* analysis, *Peason product moment correlation/Spearman’s rank-difference correlation*, *Bar graph* analysis, and *Receiver Operating Characteristics (ROC)* were used to examine the correlation between sCD26 titer variation with YS110 administration and tumor volume change, RECIST criteria evaluation and progression free survival (PFS). Mechanism for serum sCD26 titer variation was confirmed by in vitro experimentation.

**Results:**

Serum sCD26/DPP4 titer was reduced following YS110 administration and gradually recovered until the next infusion. Serum sCD26/DPP4 titer before the next infusion was sustained at lower levels in Stable Disease (SD) cases compared to Progressive Disease cases. *ROC* analysis defined the cut-off level of serum sCD26/DPP4 titer variation at day 29 pre/post for the clinical outcome of SD as tumor response or PFS. In vitro experimentation confirmed that YS110 addition reduced sCD26 production from CD26-expressing tumor and non-tumor cells.

**Conclusions:**

Our study indicates that serum sCD26/DPP4 titer variation in the early phase of YS110 treatment is a predictive biomarker for evaluating therapeutic efficacy.

**Supplementary Information:**

The online version contains supplementary material available at 10.1186/s40364-021-00273-0.

## Background

CD26 is a 110-kDa, type II transmembrane glycoprotein with dipeptidyl peptidase 4 (DPP4) activity in its extracellular domain, capable of cleaving N-terminal dipeptides with L-proline or L-alanine at the penultimate position [1, 2]. CD26 has multiple biological functions and is expressed on various normal cell types and tumors. CD26 is also found as a soluble form with conserved DPP4 activity in the serum and other body fluids. In vitro and in vivo administration of anti-CD26 mAb inhibits tumor growth, migration and invasion via multiple mechanisms of action, leading to enhanced survival of mouse xenograft models inoculated with various cancers including renal cell carcinoma (RCC) and malignant mesothelioma (MM) [3–7].

The first-in-human (FIH) phase I clinical study of YS110 for CD26-expressing solid tumors (23 MM, 9 RCC and 1 urothelial carcinoma (UTC)) was recently conducted [8], demonstrating that YS110 therapy exhibited a favorable safety profile and resulted in encouraging disease control in patients with advanced/refractory tumors.

Biomarkers in cancer management may be used for the prevention, diagnosis, and selection of therapeutic method, as well as for treatment monitoring potentially. Such markers as EGFR or ALK fusion gene (lung cancer), HER2 (breast or gastric cancer), or RAS (colon cancer) are used to select optimal therapy by identifying selected genetic alteration. However, no serum biomarker indicating a predictive outcome during a course of cancer treatment has been heretofore identified.

Serum level of soluble CD26 (sCD26) has been previously evaluated as a potential biomarker. A correlation between baseline serum sCD26 titer and clinical effectiveness of therapy has been described for patients with urothelial, gastric, pancreatic, thyroid, or lung cancer [9–14]. Serum sCD26 titer variation after colon cancer surgery was also reported to be a predictive biomarker for risk of recurrence or metastasis [15–17]. In addition, treatment with the DPP4 inhibitor sitagliptin after surgery for colorectal or lung cancer in patients with diabetes was associated with greater overall survival than treatment with other diabetic medications [18], suggesting that sCD26/DPP4 may have a role in regulating anti-tumor activity. However, there has been no report of serum sCD26 titer variation during a course of therapy being a prognostic marker of treatment outcome.

In the phase I FIH clinical trial with the humanized antibody YS110 for patients with CD26-expressing tumors, a transient decrease followed by subsequent recovery of serum sCD26/DPP4 titer level was observed during the 4-week period of the first cycle of YS110 administration. In the present study, the correlation between variation in sCD26/DPP4 titer and efficacy metrics as determined by response by RECIST criteria or progression free survival (PFS) was analyzed in a total of 26 evaluable cases or in stratified groups, to identify a potential prognostic biomarker for YS110 therapy.

## Materials and methods

### Human subjects

In the FIH phase I clinical trial, 33 patients (23 MM, 9 RCC and 1 UTC) who received YS110 were included in the safety analysis, and 26 out of 33 patients (19 MM, 6 RCC and 1 UTC) were evaluable for treatment efficacy, as described previously [8]. To determine the maximum tolerated dose, patients initially received a total of three YS110 infusions on days 1, 15 and 29 (once every 2 weeks, Q2W) at 0.1, 0.4, 1 and 2 mg/kg. On the basis of preliminary pharmacokinetics data, the protocol was then subsequently amended to allow patients to receive a total of five YS110 infusions on days 1, 8, 15, 22 and 29 (once every week, Q1W) at 2, 4 and 6 mg/kg. Among 33 patients, 26 patients (18 and 8 cases in Q2W and Q1W cohorts, respectively) were evaluable for YS110-mediated anti-tumor activity by RECIST criteria or PFS monitoring. Tumor volume variation from baseline was evaluated by a modified RECIST criteria for MM, or by RECIST 1.0 criteria for RCC or UTC on day 43 ± 4.2, two weeks following the completion of the first cycle of YS110 administration on day 29 [8]. Serum sCD26/DPP4 titer was measured immediately prior to and following YS110 administration on days 1, 15 and 29.

### Statistical analyses

*Box and Whisker plot* analysis was employed to observe variation of serum sCD26/DPP4 titer pre/post YS110 infusion on day 1, 15 and 29. *Scatter plot* analysis stratified for Stable Disease (SD) and Progressive Disease (PD) cases was employed to observe a relationship between variation of serum sCD26 titer pre/post YS110 administration on day 1, 15 and 29 and tumor volume variation from baseline on day 43. These two observational analyses then led to the usage of *PPMC* or *SRDC* analysis for the statistical examination of potential correlation between serum sCD26 titer variation from baseline pre/post YS110 administration on days 1, 15 and 29 and tumor volume variation by RECIST criteria on day 43, and PFS. Based on *Pearson product moment correlation/Spearman’s rank-difference correlation (PPMC/SRDC)* analyses, *Bar graph* analysis of the variation of serum sCD26/DPP4 titer as stratified by SD and PD cases on day 1pre (baseline, 100%), 15pre and 29pre of YS110 administration was performed to examine for correlation between serum sCD26/DPP4 variation and the incidence of SD or PD cases by RECIST criteria on day 43 with *Wilcoxon’s rank sum test*. Based on results from *PPMC/SRDC and Bar graph* analyses, *Receiver Operating Characteristics (ROC)* analysis was employed to examine the Index (cut-off titer) of serum sCD26 titer variation from baseline for the Outcome of SD by RECIST criteria, PFS ≥ 90, or ≥ 180 days, with *Fisher’s exact test*. Difference in background factors between SD and PD cases was examined by *Fisher’s exact test* or *Wilcoxon rank sum test* prior to *ROC* analysis.

### Cell lines and cultures

Human MM cell lines MSTO-211H (MSTO parent) and NCI-H226 were obtained from the American Type Culture Collection (ATCC, Rockville, MD). MSTO parent cells were stably transfected with a full-length human CD26 (MSTO-CD26) [6]. Human MM cell line JMN cells were transduced with the short hairpin RNA (shRNA)-expressing lentivirus, generating the stable cell lines JMN CD26-shRNA and JMN ctrl-shRNA [19]. For non-tumor human cells, immortalized pleural mesothelial cell line MeT-5A, mammary epithelial cell line MCF10A, fetal lung fibroblast cell line TIG-1, human umbilical vein endothelial cells (HUVEC), and human dermal microvascular endothelial cells (HDMVEC) were used. MeT-5A and MCF10A were obtained from ATCC. TIG-1 was obtained from JCRB Cell Bank (Osaka, Japan). HUVEC, HDMVEC and the culture media for MCF10A, HUVEC, HDMVEC (MEGM, EGM-2, EGM-2MV, respectively) were purchased from LONZA (Walkersville, MD). MSTO parent, MSTO-CD26, JMN ctrl-shRNA, JMN CD26-shRNA, H226 and MeT-5A were grown in RPMI 1640 medium supplemented with 10% FBS. TIG-1 was grown in DMEM medium supplemented with 10% FBS. All the cells were cultured at 37 °C in a humidified 5% CO_2_ incubator.

### Abs and reagents

Humanized anti-CD26 mAb YS110 was provided by Y’s AC Co., Ltd. (Tokyo, Japan) [5]. Human IgG_1_ isotype control mAb (clone QA16A12) purchased from BioLegend (San Diego, CA) was used as a control.

### Preparation of culture supernatant

Cells were cultured in 500 μl of culture medium in 24-well plates (Corning) in the presence or absence of control human IgG or YS110 for 3 days at 37 °C. For time-course analysis, MSTO-CD26 (1.5 × 10^5^, 4 × 10^4^, or 4 × 10^3^) were cultured in 500 μl of RPMI 1640 medium in 24-well plates in the presence or absence of YS110 (1, 3, 10 μg/ml) at 37 °C for 1, 3, or 7 days, respectively. After incubation, supernatants were collected from confluent cultures.

### Quantification of soluble CD26 and DPP4 enzyme activity

Assays for soluble CD26 and DPP4 activity were developed in our laboratory utilizing mouse anti-human CD26 mAbs (clone 5F8 and 9C11) which exhibit no cross-reactivity with the therapeutic humanized anti-CD26 mAb YS110. The relevant experimental methods were detailed previously [20]. Data were analyzed by one-way ANOVA test with Tukey’s for multiple comparison testing. Significance was analyzed using GraphPad Prism 6 (GraphPad Software, San Diego, CA). Values of *p* < 0.01 were considered significant and are indicated in the corresponding figures and figure legends.

## Results

### Changes in levels of serum sCD26/DPP4 titer pre/post YS110 administration, as documented by *Box and Whisker plot*

Several crucial parameters were included in this phase I trial such as 1) tumor histology: 19 MM, 6 RCC and 1 UTC; 2) YS110 dose: 0.1–6 mg/kg; 3) frequency of drug administration: once every 2 weeks (Q2W) for three doses in 18 cases, once every week (Q1W) for five doses in 8 cases. In addition, examination of background factors between SD and PD cases indicated that no bias was found in age, BMI, absolute value of tumor volume or serum sCD26/DPP4 titer before YS110 administration, except for gender (data not shown). In contrast to male patients (4 SD and 7 PD in MM, and 2 SD and 3 PD in RCC), YS110 appeared to be more effective in female patients (6 SD and 2 PD in MM, 1 SD in RCC, and 1PD in UTC), as shown in Additional file 1 (Tables S1) and file 2 (Table S2). Since the number of cases in each antibody dose cohort was not sufficient for statistical analysis, in the present study, a total of 26 cases were further categorized by 1) tumor histology and 2) frequency of drug administration, to examine whether serum sCD26 titer variation can be a prognostic biomarker for YS110 treatment. Detailed information regarding these 26 cases is shown in Additional file 1 (Tables S1).

**Fig. 1:**
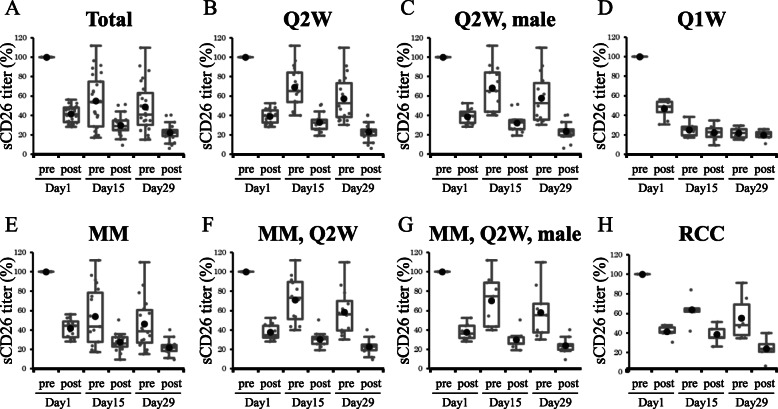
Changes in serum sCD26 levels following YS110 administration by *Box and Whisker plot* analysis. Each plot indicated the serum sCD26 titer variation from baseline (day 1pre, 100%) to pre/post of YS110 administration on days 1, 15 and 29. Analyzed data were stratified into **a** total 26 cases, **b** 18 cases with Q2W administration, **c** 14 male cases with Q2W administration, **d** 8 cases with Q1W administration, **e** 19 MM cases, **f** 12 MM cases with Q2W administration, **g** 9 MM, male cases with Q2W administration, **h** 6 RCC cases. Data are shown as mean ± S.D. in each group

We first examined serum sCD26 titer variation during a course of YS110 treatment in each group by *Box and Whisker plot* analysis. Serum sCD26 titer was consistently reduced immediately following YS110 administration on day 1, 15, 29, and gradually recovered until the next YS110 infusion, although it never returned to its former pre-dosing level (Fig. 1a). This pattern was similarly observed in the 18 cases treated on the Q2W drug administration schedule (Fig. 1b). In contrast, a clear difference was observed in the 8 cases treated on the Q1W schedule. As shown in Additional file 1 (Tables S1), relatively high antibody dose (2–6 mg/kg) was administered in the Q1W cases as compared with the Q2W cases (0.1–2 mg/kg). These differences in antibody dose and administration frequency strongly affected the serum sCD26 titer on day 15pre and day 29pre (Fig. 1d). Recovery of serum sCD26 titer following YS110 administration was not clearly observed with the more frequent drug administration of the Q1W cases. Fourteen male cases and 4 female cases received Q2W administration, while 2 male cases and 6 female cases received Q1W administration (Additional file 2 (Table S2)). The distribution bias between the male cases with Q2W and Q1W administration and the female cases with Q2W and Q1W administration was significant (*p* = 0.026 by Fisher’s exact test). In addition, the number of cases in the Q1W cohort (8 cases) was not sufficient for additional statistical analysis. Therefore, we mainly focused on the Q2W cases and the male cases for additional analyses. The initial fall and subsequent recovery of serum sCD26 titer were similarly observed in both the 19 MM cases and 6 RCC cases (Fig. 1e and h), including upon further stratification of the groups into such cohorts as the 14 male cases with Q2W administration, 12 MM cases with Q2W administration, and 9 male MM cases with Q2W administration (Fig. 1c, f and g). As shown in Additional file 3 (Fig. S1), the absolute value or titer variation of serum sCD26 titer was strongly correlated with level of serum DPP4 enzyme activity (r = 0.908, *p* < 0.001 or r = 0.974, *p* < 0.001, respectively). Since YS110 does not directly inhibit DPP4 enzyme activity [21], reduction of serum DPP4 enzyme activity following YS110 administration is therefore due to decreased serum sCD26 protein level.

### Differences in serum sCD26/DPP4 titer variation on day 29pre and tumor volume variation on day 43 for SD and PD cohorts by *Scatter plot* analysis

**Fig. 2:**
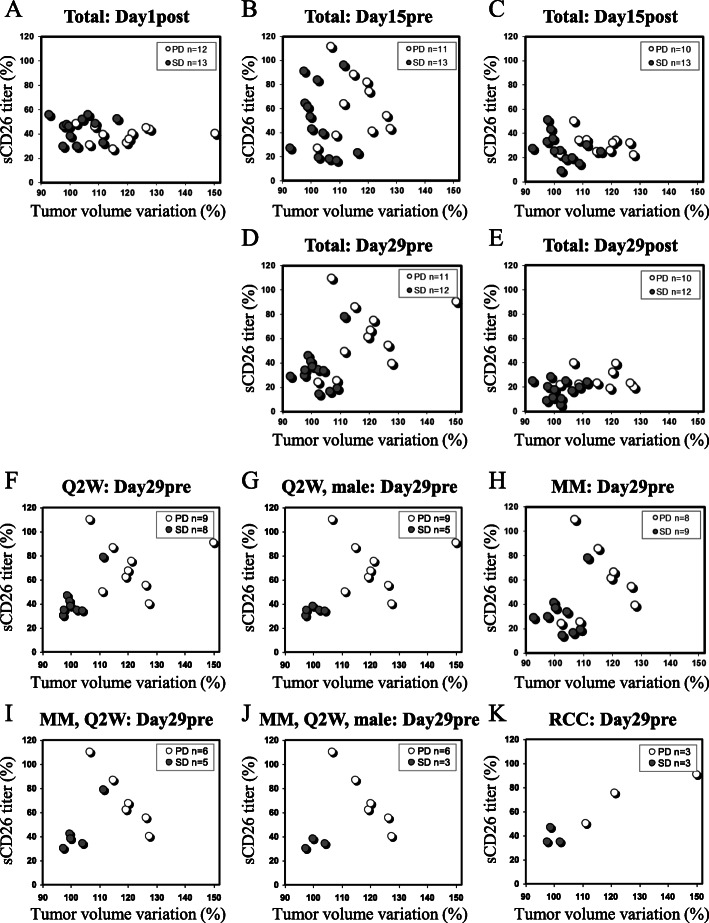
Relationship between serum sCD26 titer variation and tumor volume variation by *scatter plot* analysis. The serum sCD26 titer variation from baseline (day 1pre, 100%) on **a** day 1post, **b** day 15pre, **c** day 15post, **d** day 29pre, **e** day 29post of YS110 administration, and tumor volume variation by RECIST response criteria on day 43 of a total of 25 cases was plotted. Data were separated into SD (gray circle) and PD (white circle) cohorts. The serum sCD26 titer variation from baseline on day 29pre of YS110 administration, and tumor volume variation by RECIST response criteria on day 43 of **f** 17 cases with Q2W administration, **g** 14 male cases with Q2W administration, **h** 18 MM cases, **i** 11 MM cases with Q2W administration, **j** 9 MM, male cases with Q2W administration, **k** 6 RCC cases with Q2W administration was plotted

We next investigated a potential relationship between pre/post serum sCD26 titer variation on days 1, 15 and 29, and tumor volume variation on day 43 by *Scatter plot* analysis after the start of YS110 administration, with a total of 25 cases stratified by SD and PD cohorts. The tumor volume variation of the SD group would be expected to naturally be lower than that of the PD group. Serum sCD26 titers were markedly decreased in both SD and PD cohorts immediately post YS110 infusion on days 1, 15 and 29 (Fig. 2a, c and e). On the other hand, a noticeable difference between the SD and PD groups in the serum sCD26 titer variation was observed on day 29pre. Serum sCD26 titer variation on day 29pre of the SD cohort was at a lower level compared with the PD group (Fig. 2d). Moreover, this phenomenon was clearly observed in each stratified group such as the 17 cases with Q2W administration, 14 male cases with Q2W administration, 18 MM cases, 11 MM cases with Q2W administration, 9 male MM cases with Q2W administration, or 6 RCC cases (Fig. 2f-k, respectively). These *Scatter plot* analyses indicate that the serum sCD26 titer variation of the SD cohort was lower than that of the PD cases when measured prior to YS110 administration, and the difference was particularly evident on day 29pre with the Q2W administration.

### Correlation of pre/post serum sCD26/DPP4 titer variation on day 29 with tumor volume variation and/or PFS by *PPMC/SRDC* analyses

*PPMC* and *SRDC* analyses were conducted to examine the correlation between pre/post serum sCD26 titer variation on days 1, 15 and 29, and tumor volume variation as determined by RECIST criteria at day 43 after YS110 administration or PFS. In the FIH phase I clinical trial, 13 cases were assessed as SD and 13 cases were assessed as PD by RECIST, and among the 13 SD cases, YS110 was particularly effective in 7 cases with PFS being longer than 180 days (Additional file 1 (Tables S1)). In a total of 25 cases, statistically significant correlation between day 29pre serum sCD26 titer variation and tumor volume variation on day 43 was observed (*p* = 0.006 or *p* = 0.009 by *PPMC*/*SRDC*, respectively (Table 1). There was also statistically significant correlation between serum sCD26 titer variation and PFS (*p* = 0.011 by *PPMC* on day 29post for a total 26 cases (Table 1). In addition, there was statistically significant correlation between variation in serum titer of DPP4 enzymatic activity and tumor volume or PFS, similar to the case with serum sCD26 titer (Table 1). Statistically significant correlation was similarly observed in variation between day 29pre serum sCD26/DPP4 titer and tumor volume, and between pre and/or post day 29 serum sCD26/DPP4 titer and PFS in 18 cases with Q2W administration frequency and 14 male cases with Q2W administration frequency (Additional file 4 (Table S3) and file 5 (Table S4)). In 19 MM cases, statistically significant correlation between variation in pre/post day 29 serum DPP4 titer and tumor volume was observed by *SRDC* analysis, while the correlation between day 29pre serum sCD26 titer and tumor volume almost reached statistical significance (*p* = 0.065) by *PPMC* analysis. There was statistically significant correlation between day 29post serum sCD26 titer and PFS by *PPMC* analysis, while the correlation between day 29post serum DPP4 titer and PFS almost reached statistical significance (*p = 0.056* by *PPMC* or *p* = 0.069 by *SRDC* analysis) (Additional file 6 (Table S5)). In 12 MM cases with Q2W administration frequency, no statistically significant correlation between variation of serum sCD26/DPP4 titer and tumor volume was observed. The correlation between day 29post serum sCD26/DPP4 titer and PFS did reach statistical significance (Additional file 7 (Table S6)). In the 9 male MM cases treated with Q2W administration, no significant difference was observed in variation between serum sCD26/DPP4 titer and tumor volume, although there was a trend for a correlation between pre/post day 29 serum sCD26/DPP4 titer and PFS (Additional file 8 (Table S7)). In the 6 RCC cases and 8 cases treated with Q2W and Q1W administration respectively, the number of cases were not enough for *PPMC/SRDC* statistical analysis. These results indicate that there was a correlation in variation between pre/post day 29 serum sCD26/DPP4 titer (before/after the third YS110 administration) and tumor volume or PFS. Importantly, statistical significance was reached although there was limited number of cases with each stratified cohort, particularly in the 18 cases and 14 male cases treated with Q2W administration.

**Table 1:** Correlation between serum sCD26/DPP4 titer variation and tumor volume change/PFS by PPMC or SRDC analysis

			Peason’ s Product -moment correlation		Spearman’ s rank difference correlation	
Var .1	Var .2	*n*	r	*P* value	ρ	*P* value
tumor volume %change	PFS	25	- 0. 514	0. 008 **	- 0. 504	0. 014 *
tumor volume %change	sCD26 Day 1 Post	25	- 0. 214	0. 308	- 0. 198	0. 333
〃	sCD26 Day15 Pr e	24	- 0. 085	0. 696	0. 002	0. 993
〃	sCD26 Day15 Post	23	- 0. 115	0. 606	- 0. 169	0. 428
〃	sCD26 Day29 Pr e	23	0. 548	0. 006 *	0. 553	0. 009 **
〃	sCD26 Day29 Post	22	0. 358	0. 102	0. 304	0. 163
〃	DPP4 Day 1 Post	25	- 0. 146	0. 490	- 0. 182	0. 374
〃	DPP4 Day15 Pr e	24	- 0. 068	0. 757	- 0. 023	0. 910
〃	DPP4 Day15 Post	23	- 0. 039	0. 862	- 0. 037	0. 864
〃	DPP4 Day29 Pr e	23	0. 502	0. 014	0. 531	0. 013 *
〃	DPP4 Day29 Post	22	0. 379	0. 082	0. 451	0. 039 *
PFS (days)	sCD26 Day 1 Post	26	0. 047	0. 821	0. 083	0. 678
〃	sCD26 Day15 Pr e	25	- 0. 021	0. 922	- 0. 099	0. 626
〃	sCD26 Day15 Post	24	- 0. 010	0. 964	0. 017	0. 935
〃	sCD26 Day29 Pr e	24	- 0. 351	0. 093	- 0. 205	0. 325
〃	sCD26 Day29 Post	23	- 0. 521	0. 010 **	- 0. 332	0. 119
〃	DPP4 Day 1 Post	26	- 0. 109	0. 600	- 0. 048	0. 809
〃	DPP4 Day15 Pr e	25	- 0. 022	0. 919	- 0. 089	0. 663
〃	DPP4 Day15 Post	24	- 0. 034	0. 877	- 0. 072	0. 732
〃	DPP4 Day29 Pr e	24	- 0. 253	0. 235	- 0. 167	0. 423
〃	DPP4 Day29 Post	23	- 0. 442	0. 034 *	- 0. 381	0. 074

### Day29pre serum sCD26/DPP4 titer of SD cohort was significantly lower than that of PD cohort by *Bar graph* analysis

**Fig. 3:**
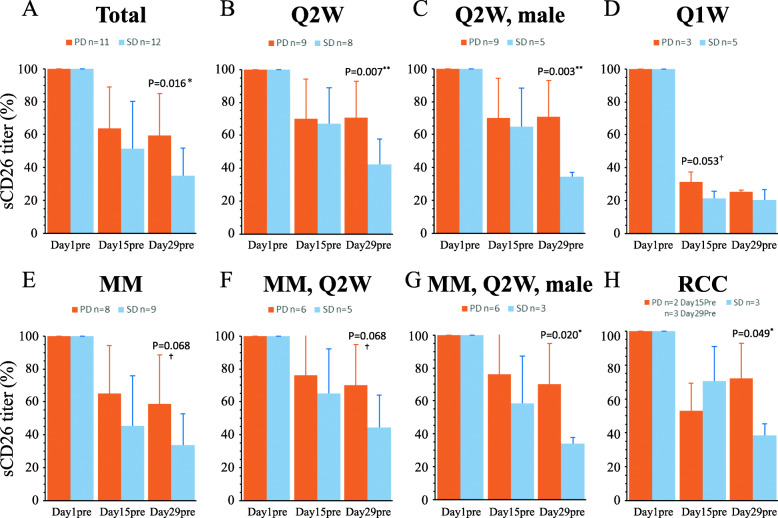
Difference in serum sCD26 titer variation between SD and PD cohorts by *bar graph* analysis. The difference of serum sCD26 titer variation from baseline (day 1pre, 100%) on day 1pre, day 15pre and day 29pre of YS110 administration between SD and PD cohorts was analyzed. Analyzed data were stratified into **a** total 23 cases, **b** 17 cases with Q2W administration, **c** 14 male cases with Q2W administration, **d** 8 cases with Q1W administration, **e** 17 MM cases, **f** 11 MM cases with Q2W administration, **f** 9 MM, male cases with Q2W administration, **h** 6 RCC cases with Q2W administration. Data are shown as mean ± S.D. in each group

Based on scatter plot and *PPMC/SRDC* examination, *Bar graph* analysis of day 1pre, 15pre and 29pre serum sCD26 titer variation in SD and PD cases was conducted. Of the total 23 cases (12 SD and 11 PD), serum sCD26 titers of both SD and PD cohorts were reduced from day 1pre to day 29pre samples. Of note, day 29pre serum sCD26 titer variation of SD cases was significantly lower than that of PD cases (*p* = 0.016) (Fig. 3a). Similar results were observed for each stratified group such as the 17 cases treated with Q2W administration (*p* = 0.007), 17 MM cases (*p* = 0.068), 11 MM cases treated with Q2W administration (*p* = 0.068), 9 male MM cases treated with Q2W administration (*p* = 0.020), or 6 RCC cases (*p* = 0.049) (Fig. 3b and e-h). Statistically significant difference between the SD and PD cohorts with the smallest *p*-value was observed in the 14 male cases treated with Q2W administration (*p* = 0.003) (Fig. 3c). In the 8 cases treated with Q1W administration, serum sCD26 titer variation of SD cases was lower than that of PD cases, trending toward statistical significance (*p* = 0.053) on day15pre prior to the third YS110 administration, which represented the same timing for sample collection to evaluate day 29pre serum sCD26 titer in the Q2W treatment schedule (Fig. 3d).

### Predictive power of serum sCD26/DPP4 titer variation on outcomes of SD or PFS by *ROC* analysis in the stratified groups

*ROC* analysis was employed to determine the cut-off titer (the Index) of serum sCD26/DPP4 titer variation on day 29pre/post YS110 administration for the Outcomes of SD and PFS ≥ 90 or ≥ 180 days. Probability was evaluated by *Fisher*^*’*^*s exact test* (Table 2). A total of 23 cases was examined to determine the Index (46.4% or 18.2%) for the Outcomes SD, PFS ≥ 90 or ≥ 180 days, with statistically significant results (*p* = 0.003 for SD, and 0.005 or 0.003 for PFS, respectively, and with Area Under the Curve (AUC) 0.795, 0.697 or 0.759, respectively) (Table 2; Column Total). For Column Q2W (17 or 18 cases), Q2W, males (14 cases), MM (17 or 18 cases), MM, Q2W (11 or 12 cases), and MM, Q2W, males (9 cases), the Indexes of each column for the Outcomes were determined to have statistical significance or tendency toward significance. Particularly for Column Q2W, males (14 cases), the Index 37.7% on day29pre YS110 administration for the Outcome SD was statistically significant (*p* < 0.001 with AUC 1.000). Also, the Index 37.7% for the Outcome PFS ≥ 90 or ≥ 180 days was statistically significant (*p* < 0.001 or *p* = 0.027, and with AUC 0.950 or 0.879, respectively). Taken together, our analyses of serum sCD26/DPP4 titer variation during a course of YS110 treatment demonstrate that serum sCD26/DPP4 titer variation, particularly at the time point immediately prior to/following the third YS110 infusion on Day29 in the Q2W administration schedule, was a potential prognostic biomarker for YS110 anti-tumor therapy.

**Table 2:** ROC analysis

Column No.		Total	Q2W	Q2W, male	MM	MM, Q2W	MM, Q2W, male
Cases of analysis for SD or PFS Outcome		23 cases (SD/PFS)	17 Cases (SD) 18 Cases (PFS)	Male 14 Cases (SD / PFS)	Malignant Mesothelioma 17 Cases (SD) 18Cases (PFS)	Malignant Mesothelioma 11 Cases (SD) 12 Cases (PFS)	Malignant Mesothelioma Male 9 Cases (SD/PFS)
Cases with Administration Frequency	Cases with Q2W	17	17 or 18	14	11 or 12	11 or 12	9
	Cases with Q1W	6	0	0	6	0	0
Outcome: SD Index: Cut-off titer with variation of serum sCD26(%) titer from baseline	sCD26 measured point	Day29 Pre	Day29 Pre	Day29 Pre	Day29 Post	Day29 Post	Day29 Pre
	Index: Cut-off values	46.4	46.4	37.7	18.2	18.2	37.7
	AUC	0.795	0.888	1.000	0.736	0.733	1.000
	Sensitivity(%)	91.7 (11/12)	87.5 (7/8)	100.0 (5/5)	55.6 (5/9)	60.0 (3/5)	100.0 (3/3)
	1-Specificity(%)	27.3 (3/11)	11.1 (1/9)	0.0 (0/9)	0.0 (0/8)	0.0 (0/6)	0.0 (0/6)
	Fisher’s Exact Test	P = 0.003 **	P = 0.003 **	P < 0.001 **	*P* = 0.029 *	*P* = 0.061 †	*P* = 0.012 *
	PPV	0.786	0.875	1.000	1.000	1.000	1.000
	NPV	0.889	0.889	1.000	0.667	0.750	1.000
Outcome: PFS > 90 Index: Cut-off titer with variation of serum sCD26(%) titer from baseline	sCD26 measured point	Day29 Post	Day29 Pre	Day29 Pre	Day29 Post	Day29 Post	Day29 Pre
	Index: Cut-off values	18.2	46.4	37.7	18.2	18.2	37.7
	AUC	0.692	0.917	0.950	0.708	0.812	1.000
	Sensitivity(%)	62.5 (5/8)	100.0 (6/6)	100.0 (4/4)	66.7 (4/6)	75.0 (3/4)	100.0 (3/3)
	1-Specificity(%)	6.7 (1/15)	16.7 (2/12)	10.0 (1/10)	8.3 (1/12)	0.0 (0/8)	0.0 (0/6)
	Fisher’s Exact Test	P = 0.009 **	*P* = 0.002 **	P < 0.001 **	*P* = 0.022 *	*P* = 0.018 *	P = 0.012 *
	PPV	0.833	0.750	0.800	0.800	1.000	1.000
	NPV	0.824	1.000	1.000	0.846	0.889	1.000
Outcome: PFS > 180 Index: Cut-off titer with variation of serum sCD26(%) titer from baseline	sCD26 measured point	Day29 Post	Day29 Pre	Day29 Pre	Day29 Post	Day29 Post	Day29 Pre
	Index: Cut-off values	18.2	46.4	37.7	18.2	18.2	37.7
	AUC	0.759	0.846	0.879	0.815	1.000	0.929
	Sensitivity(%)	71.4 (5/7)	100.0 (5/5)	100.0 (3/3)	80.0 (4/5)	100.0 (3/3)	100.0 (2/2)
	1-Specificity(%)	6.3 (1/16)	23.1 (3/13)	18.2 (2/11)	7.7 (1/13)	0.0 (0/9)	14.3 (1/7)
	Fisher’s Exact Test	P = 0.003 **	P = 0.007 **	P = 0.027 *	*P* = 0.008 **	*P* = 0.005 **	*P* = 0.083 †
	PPV	0.833	0.625	0.600	0.800	1.000	0.667
	NPV	0.882	1.000	1.000	0.923	1.000	1.000

### Addition of humanized anti-CD26 mAb reduced sCD26 levels in culture supernatants of CD26-expressing MM cell lines and non-tumor cells

**Fig. 4:**
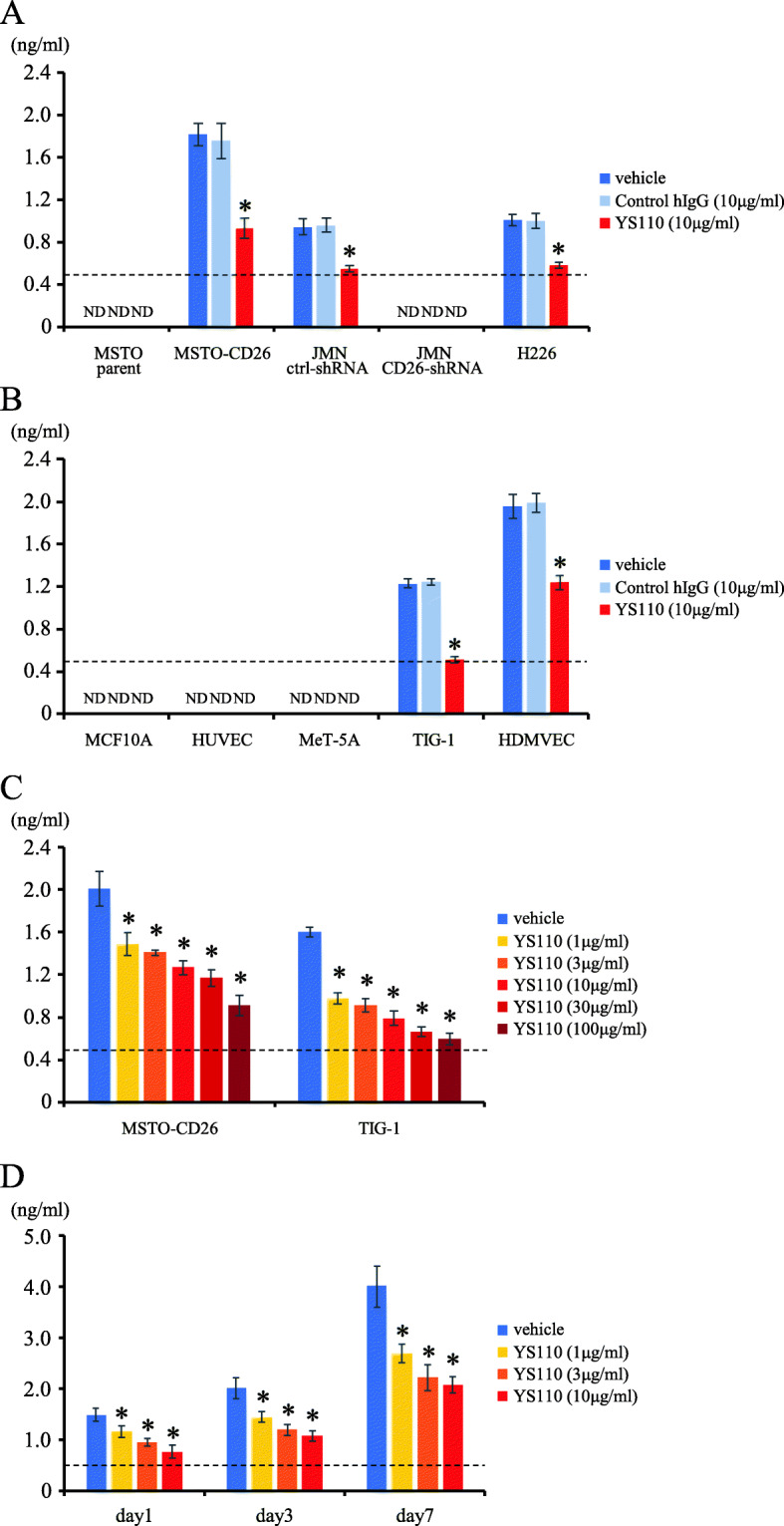
Addition of YS110 reduced the soluble CD26 production from CD26-positive tumor and non-tumor cells. **a**, **b** MM cell lines (MSTO parent, MSTO-CD26, JMN ctrl-shRNA, JMN CD26-shRNA or H226 cells (3.5 × 10^4^, each)) **a** or non-tumor cells (MCF10A (1.0 × 10^5^), HUVEC (9.0 × 10^4^), MeT-5A (6.0 × 10^4^), TIG-1 (5.0 × 10^4^) or HDMVEC cells (9.0 × 10^4^)) **b** were incubated with control human IgG (hIgG) or the humanized anti-CD26 mAb YS110 (10 μg/ml, each) for 72 h. **c** MSTO-CD26 or TIG-1 cells were incubated with the indicated concentrations of YS110 for 3 days. **d** MSTO-CD26 cells were incubated with the indicated concentrations of YS110 for 1 day, 3 days or 7 days. Concentrations of soluble CD26 in the culture supernatants were examined by ELISA. The dashed line indicates the detection limit (0.488 ng/ml), and ND denotes ‘not detected’ (under detection limit). Representative data of three independent experiments are shown as mean ± S.D. of quadruplicate samples, comparing values with YS110 to those with vehicle or control human IgG (* *p* < 0.01), and similar results were obtained in each experiment

Since sCD26 serum levels were markedly decreased in patients with CD26-expressing tumors following YS110 treatment in the phase I study (Fig. 1), we investigated the in vitro effect of YS110 on sCD26 production from MM cell lines. For this purpose, we selected various human CD26-positive or negative MM cell lines. MSTO parent was an endogenous human CD26-deficit cell line, while MSTO-CD26 stably expressed a full-length human CD26 [6]. Stable shRNA knockdown of CD26 in JMN, an endogenous human CD26-positive cell line, markedly reduced CD26 expression as compared with JMN ctrl-shRNA cells [19]. Cell surface expression of CD26 on MM cell lines was shown in Additional file 9 (Fig. S2a). We first measured the amount of sCD26 contained in the culture supernatants from a 3-day culture of CD26-positive or negative cells. sCD26 could be quantified in the culture supernatants of CD26-positive MSTO-CD26, JMN ctrl-shRNA and H226 cells, whereas sCD26 could not be detected in the culture supernatants of CD26-negative MSTO parent and JMN CD26-shRNA cells, regardless of YS110 treatment (Fig. 4a). Treatment with YS110 clearly reduced the amount of sCD26 in the culture supernatants of MSTO-CD26, JMN ctrl-shRNA and H226 cells, as compared with those cells incubated with vehicle or control human IgG (Fig. 4a). We next examined the production of sCD26 from non-tumor (normal) cells. CD26 was clearly expressed on the cell surface of HDMVEC and TIG-1, while CD26 was hardly expressed on HUVEC and MCF10A, and partially expressed on MeT-5A (Additional file 9 (Fig. S2b)). sCD26 could be quantified in the culture supernatants of CD26-positive TIG-1 and HDMVEC cells, whereas sCD26 could not be detected in the culture supernatants of CD26-negative or low MCF10A, HUVEC and MeT-5A cells (Fig. 4b). Similar with the results shown in Fig. 4a, YS110 treatment clearly reduced the amount of sCD26 in the culture supernatants of TIG-1 and HDMVEC cells, as compared with those cells incubated with vehicle or control human IgG (Fig. 4b). Treatment with YS110 resulted in decreased production of sCD26 from both MSTO-CD26 and TIG-1 cells in a dose-dependent manner (Fig. 4c). Subsequent time course analysis showed that sCD26 level in the supernatant of a 3-day culture of MSTO-CD26 cells was slightly enhanced compared to 1-day culture of MSTO-CD26 cells, and increased sCD26 level was observed in the supernatant of a 7-day culture of MSTO-CD26 cells (Fig. 4d). Reduction of sCD26 level following YS110 treatment was consistently observed at any culture period. Taken together, these data indicate that sCD26 was produced from both CD26-positive tumor cells and non-tumor cells, and the addition of YS110 reduced sCD26 production from those cells in an antibody dose-dependent manner. It is our hypothesis that these in vitro effects are reflected in the marked reduction of sCD26 level in the serum of patients with CD26-expressing tumors following YS110 administration.

## Discussion

In the present study, *scatter plot* analysis of the relationship between serum sCD26/DPP4 titer variation and tumor volume variation by RECIST response criteria suggested that a predictable time period during the course of YS110 treatment can be used to distinguish between SD and PD cases. This predictable time period was found to be day 29pre/post the third dose of YS110 administration in the Q2W treatment schedule, with results being statistically significant by *PPMC/SRDC* and *Bar graph* analyses. The *ROC* analysis defined the cut-off titer of serum sCD26/DPP4 titer variation at day 29pre/post as the Index for the outcome of cases with SD or with PFS longer than 90 or 180 days, resulting in a significantly feasible prediction under the Index obtained. In particular, *ROC* analysis of the 14 male cases treated with the Q2W schedule defined the cut-off titer with *p* < 0.001 (Table 2). Similar results were obtained in the 9 male MM cases treated with the Q2W administration schedule (Table 2). The results were statistically significant despite the small number of cases in the stratified groups, strongly suggesting that serum sCD26/DPP4 titer variation was a definitive prognostic biomarker for cancer patients treated with YS110. In cases treated with the Q1W schedule, the number of cases were not sufficient for analysis, in contrast to the situation with the Q2W schedule. However, serum sCD26 titer variation on Day15pre and not on Day29pre could be used to discriminate SD from PD cases with a trend toward statistical significance (*p* = 0.053) as shown in Fig. 3d. These data would suggest that the increase in drug administration frequency and dosage (Q1W at YS110 dose level 2, 4, 6 mg/kg) could have an effect on the optimal timing of serum sCD26 titer measurement, which can be altered depending on administration frequency and/or dosage of YS110.

Our robust in vitro and in vivo data indicated that YS110 induced cell lysis of MM cells via antibody-dependent cell-mediated cytotoxicity (ADCC) in addition to its direct anti-tumor effect through the induction of cell cycle arrest at S/G1 phase [5, 22]. Another important mechanism of action of YS110 was the nuclear translocation of CD26 molecules by internalization of the CD26-YS110 complexes from the cell surface to inhibit proliferation of MM cells via suppression of POLR2A gene expression, a component of RNA polymerase II. However, in the case of CD26-expressing non-neoplastic cells such as human embryonic kidney HEK293 cells or normal T lymphocytes, the CD26-YS110 complex was not translocated into the nucleus [23, 24]. Moreover, internalization of the CD26-antibody complexes was dependent on the epitope of CD26 recognized by specific mAb. Internalization of CD26 was not observed from the cell surface of MM cells treated with the murine anti-human CD26 mAb 5F8, which recognized a different epitope of CD26 from that recognized by YS110 and did not exert anti-tumor activity [23, 25].

Residues 201 to 211, 730 and 740 of CD26 along with the serine catalytic site at residue 630, which constitute a CD26/DPPIV pocket structure, are essential for DPP4 enzyme activity [26]. In contrast, YS110 recognizes the 248-358th aa region of CD26, which is distinct from its catalytic site [25, 27], and binding of YS110 does not directly affect DPP4 enzyme activity [21]. Our present data showed that YS110 treatment reduced the production of sCD26 from both CD26-expressing MM cell lines and non-tumor cells (Fig. 4). Although the soluble form of CD26 begins at the 39th aa residue and lacks the cytoplasmic and transmembrane regions [28], the precise mechanisms involved in sCD26 production and release from the cell surface are not yet fully elucidated. It is possible that decreased sCD26 production following YS110 treatment was due to antibody-mediated internalization of cell surface CD26 molecules [23]. In the phase I clinical trial involving YS110, serum level of sCD26 immediately following YS110 administration on day 1 (day 1post) was markedly decreased as compared with the level prior to YS110 administration (day 1pre) (Fig. 1). Fc receptor-mediated phagocytosis of sCD26-YS110 complexes by phagocytes may possibly be involved in this rapid reduction of serum sCD26 following YS110 administration. In the present study, we demonstrated that sustained low levels of serum sCD26/DPP4 titer following YS110 administration was commonly observed in SD cases compared with those in PD cases, while there was no significant difference in the serum sCD26/DPP4 levels immediately after YS110 administration (days 1post, 15post and 29post) between SD and PD cases (Figs. 1 and 2). Future research is required to identify the factors involved in the retention or restoration of serum sCD26/DPP4 levels after YS110 administration.

In addition to the mechanisms of action responsible for the anti-tumor activity of YS110 as described above, recent works demonstrated the functional role of DPP4-mediated post-translational modification of chemokines in regulating tumor immunity through its interaction with its substrates. The exact chemokines produced at the tumor microenvironment (TME) are different, depending on tumor histology. In vivo tumor-transplant models showed that the DPP4 inhibitor sitagliptin reduced tumor growth through the preservation of bioactive CXCL10 in the TME of melanoma and colon carcinoma. In the normal physiological state, CXCL10 is rapidly degraded by DPP4, resulting in decreased recruitment and migration of CXCR3^+^ T cells and NK cells into the TME. In contrast, DPP4 inhibition enhanced tumor rejection by preserving the full-length biologically active form of CXCL10, leading to increased trafficking of CXCR3^+^ cells into the TME [29, 30]. Similar with CXCL10, administration of sitagliptin resulted in higher concentrations of bioactive CCL11 in the TME of hepatocellular carcinoma and breast cancer, leading to increased migration of eosinophils into solid tumors. In these models, expression of IL-33 in tumors was a key inducer of CCL11 production and eosinophil-mediated anti-tumor responses [31]. In view of these findings, our data showing that serum DPP4 activity was decreased following YS110 treatment would suggest enhancement of tumor immunity via DPP4 inhibition may constitute yet another mechanism of action of YS110.

## Conclusions

This is the first finding that the serum sCD26/DPP4 titer variation in the early phase of treatment with the humanized anti-CD26 antibody YS110 may be a predictive biomarker for anti-tumor activity for patients with CD26^+^ cancers including MM. Future clinical trials involving a larger group of patients would be needed for further validation of the predictive/prognostic value of serum sCD26 in patients treated with YS110.

## Supplementary Information

**Additional file 1: Table S1.** Demographic chart of 26 evaluable cases on administration frequency and dosage, gender, PFS, age, BMI, RECIST evaluation, tumor volume change and serum sCD26/DPP4 titer change

**Additional file 2: Table S2.** Detailed information about 26 evaluable cases

**Additional file 3: Figure S1.** Correlation between serum soluble CD26 level and DPP4 enzyme activity

**Additional file 4: Table S3.** Correlation between serum sCD26/DPP4 titer variation (%) and tumor volume change (%) or PFS (days) in 18 cases with Q2W administration by PPMC or SRDC analysis

**Additional file 5: Table S4.** Correlation between serum sCD26/DPP4 titer variation (%) and tumor volume change (%) or PFS (days) in 14 male cases with Q2W administration by PPMC or SRDC analysis

**Additional file 6: Table S5.** Correlation between serum sCD26/DPP4 titer variation (%) and tumor volume change (%) or PFS (days) in 19 MM cases by PPMC or SRDC analysis.

**Additional file 7: Table S6.** Correlation between serum sCD26/DPP4 titer variation (%) and tumor volume change (%) or PFS (days) in 12 MM cases with Q2W administration by PPMC or SRDC analysis.

**Additional file 8: Table S7.** Correlation between serum sCD26/DPP4 titer variation (%) and tumor volume change (%) or PFS (days) in 9 MM, male cases with Q2W administration by PPMC or SRDC analysis.

**Additional file 9: Figure S2.** Cell surface protein expression of CD26 on the human tumor and non-tumor cells.

## Data Availability

All data generated or analyzed during this study are included in this article and its supplementary information files.

## Abbreviations

AUC: Area under the curve
DPP4: Dipeptidyl peptidase 4
FIH: First-in-human
HDMVEC: Human dermal microvascular endothelial cells
HUVEC: Human umbilical vein endothelial cells
mAb: Monoclonal antibody
MM: Malignant mesothelioma
PD: Progressive disease
PFS: Progression free survival
PPMC: Pearson product moment correlation
Q1W: Once every week
Q2W: Once every 2 weeks
RCC: Renal cell carcinoma
ROC: Receiver operating characteristics
sCD26: Soluble CD26
SD: Stable disease
shRNA: Short hairpin RNA
SRDC: Spearman’s rank-difference correlation
TME: Tumor microenvironment
UTC: Urothelial carcinoma
