# Supplementary material for: Serum soluble CD26/DPP4 titer variation is a potential prognostic biomarker in cancer therapy with a humanized anti-CD26 antibody
Source: Biomark Res. 2021 Mar 23;9:21. doi: 10.1186/s40364-021-00273-0 (PMC7989014; doi:10.1186/s40364-021-00273-0)
Supplement: Supplementary file 1 — Additional file 1: Table S1. Demographic chart of 26 evaluable cases on administration frequency and dosage, gender, PFS, age, BMI, RECIST evaluation, tumor volume change and serum sCD26/DPP4 titer change [file 40364_2021_273_MOESM1_ESM.pptx]

## Slide 1
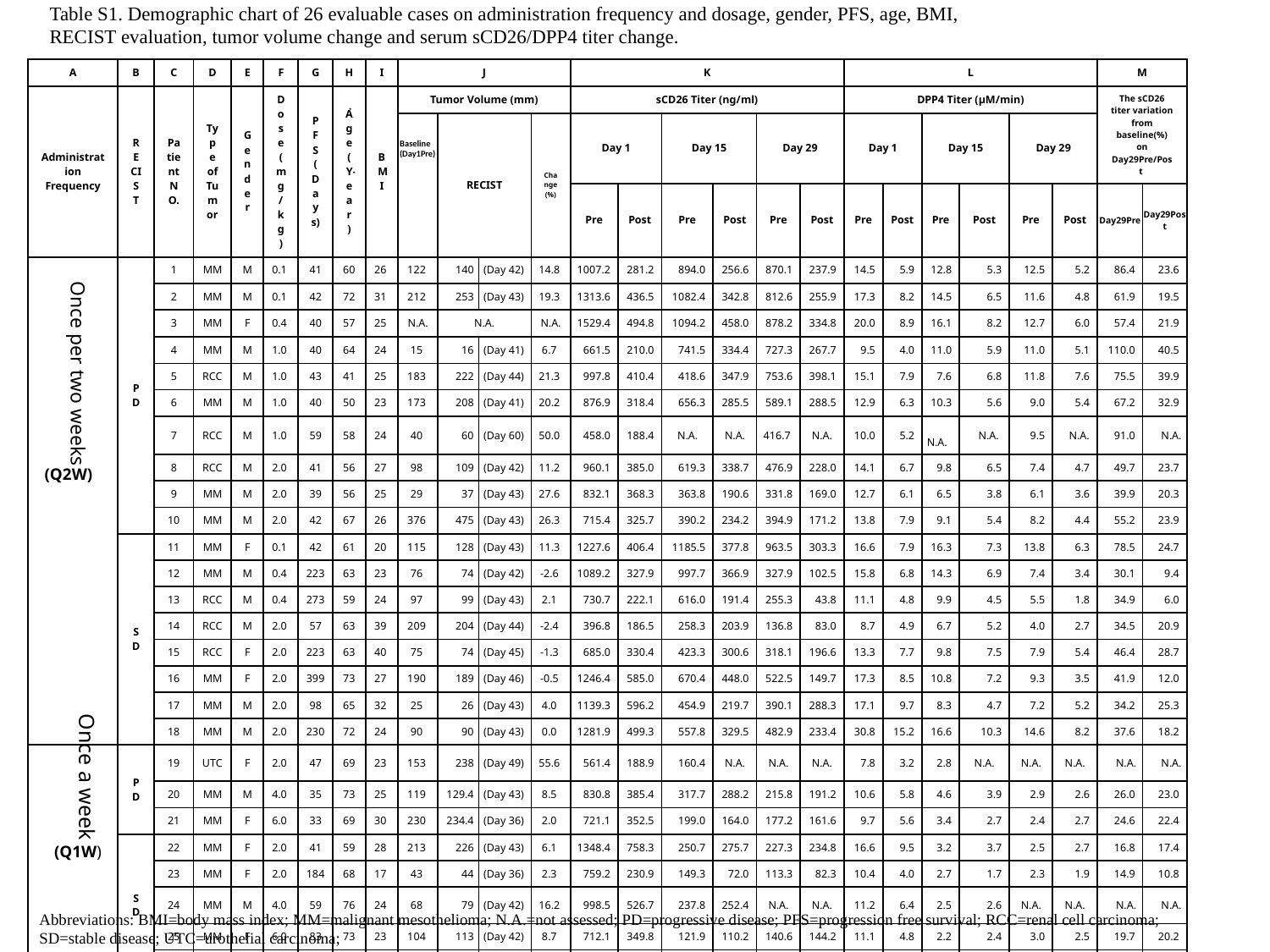

Table S1. Demographic chart of 26 evaluable cases on administration frequency and dosage, gender, PFS, age, BMI,
RECIST evaluation, tumor volume change and serum sCD26/DPP4 titer change.
| A | B | C | D | E | F | G | H | I | J | | | | K | | | | | | L | | | | | | M | |
| --- | --- | --- | --- | --- | --- | --- | --- | --- | --- | --- | --- | --- | --- | --- | --- | --- | --- | --- | --- | --- | --- | --- | --- | --- | --- | --- |
| Administration Frequency | RECIST | Patient NO. | Type of Tumor | Gender | Dose (mg/kg) | PFS (Days) | Age (Year) | BMI | Tumor Volume (mm) | | | | sCD26 Titer (ng/ml) | | | | | | DPP4 Titer (μM/min) | | | | | | The sCD26 titer variation from baseline(%) on Day29Pre/Post | |
| | | | | | | | | | | RECIST | | Change (%) | Day 1 | | Day 15 | | Day 29 | | Day 1 | | Day 15 | | Day 29 | | | |
| | | | | | | | | | | | | | Pre | Post | Pre | Post | Pre | Post | Pre | Post | Pre | Post | Pre | Post | Day29Pre | Day29Post |
| | PD | 1 | MM | M | 0.1 | 41 | 60 | 26 | 122 | 140 | (Day 42) | 14.8 | 1007.2 | 281.2 | 894.0 | 256.6 | 870.1 | 237.9 | 14.5 | 5.9 | 12.8 | 5.3 | 12.5 | 5.2 | 86.4 | 23.6 |
| | | 2 | MM | M | 0.1 | 42 | 72 | 31 | 212 | 253 | (Day 43) | 19.3 | 1313.6 | 436.5 | 1082.4 | 342.8 | 812.6 | 255.9 | 17.3 | 8.2 | 14.5 | 6.5 | 11.6 | 4.8 | 61.9 | 19.5 |
| | | 3 | MM | F | 0.4 | 40 | 57 | 25 | N.A. | N.A. | | N.A. | 1529.4 | 494.8 | 1094.2 | 458.0 | 878.2 | 334.8 | 20.0 | 8.9 | 16.1 | 8.2 | 12.7 | 6.0 | 57.4 | 21.9 |
| | | 4 | MM | M | 1.0 | 40 | 64 | 24 | 15 | 16 | (Day 41) | 6.7 | 661.5 | 210.0 | 741.5 | 334.4 | 727.3 | 267.7 | 9.5 | 4.0 | 11.0 | 5.9 | 11.0 | 5.1 | 110.0 | 40.5 |
| | | 5 | RCC | M | 1.0 | 43 | 41 | 25 | 183 | 222 | (Day 44) | 21.3 | 997.8 | 410.4 | 418.6 | 347.9 | 753.6 | 398.1 | 15.1 | 7.9 | 7.6 | 6.8 | 11.8 | 7.6 | 75.5 | 39.9 |
| | | 6 | MM | M | 1.0 | 40 | 50 | 23 | 173 | 208 | (Day 41) | 20.2 | 876.9 | 318.4 | 656.3 | 285.5 | 589.1 | 288.5 | 12.9 | 6.3 | 10.3 | 5.6 | 9.0 | 5.4 | 67.2 | 32.9 |
| | | 7 | RCC | M | 1.0 | 59 | 58 | 24 | 40 | 60 | (Day 60) | 50.0 | 458.0 | 188.4 | N.A. | N.A. | 416.7 | N.A. | 10.0 | 5.2 | N.A. | N.A. | 9.5 | N.A. | 91.0 | N.A. |
| | | 8 | RCC | M | 2.0 | 41 | 56 | 27 | 98 | 109 | (Day 42) | 11.2 | 960.1 | 385.0 | 619.3 | 338.7 | 476.9 | 228.0 | 14.1 | 6.7 | 9.8 | 6.5 | 7.4 | 4.7 | 49.7 | 23.7 |
| | | 9 | MM | M | 2.0 | 39 | 56 | 25 | 29 | 37 | (Day 43) | 27.6 | 832.1 | 368.3 | 363.8 | 190.6 | 331.8 | 169.0 | 12.7 | 6.1 | 6.5 | 3.8 | 6.1 | 3.6 | 39.9 | 20.3 |
| | | 10 | MM | M | 2.0 | 42 | 67 | 26 | 376 | 475 | (Day 43) | 26.3 | 715.4 | 325.7 | 390.2 | 234.2 | 394.9 | 171.2 | 13.8 | 7.9 | 9.1 | 5.4 | 8.2 | 4.4 | 55.2 | 23.9 |
| | SD | 11 | MM | F | 0.1 | 42 | 61 | 20 | 115 | 128 | (Day 43) | 11.3 | 1227.6 | 406.4 | 1185.5 | 377.8 | 963.5 | 303.3 | 16.6 | 7.9 | 16.3 | 7.3 | 13.8 | 6.3 | 78.5 | 24.7 |
| | | 12 | MM | M | 0.4 | 223 | 63 | 23 | 76 | 74 | (Day 42) | -2.6 | 1089.2 | 327.9 | 997.7 | 366.9 | 327.9 | 102.5 | 15.8 | 6.8 | 14.3 | 6.9 | 7.4 | 3.4 | 30.1 | 9.4 |
| | | 13 | RCC | M | 0.4 | 273 | 59 | 24 | 97 | 99 | (Day 43) | 2.1 | 730.7 | 222.1 | 616.0 | 191.4 | 255.3 | 43.8 | 11.1 | 4.8 | 9.9 | 4.5 | 5.5 | 1.8 | 34.9 | 6.0 |
| | | 14 | RCC | M | 2.0 | 57 | 63 | 39 | 209 | 204 | (Day 44) | -2.4 | 396.8 | 186.5 | 258.3 | 203.9 | 136.8 | 83.0 | 8.7 | 4.9 | 6.7 | 5.2 | 4.0 | 2.7 | 34.5 | 20.9 |
| | | 15 | RCC | F | 2.0 | 223 | 63 | 40 | 75 | 74 | (Day 45) | -1.3 | 685.0 | 330.4 | 423.3 | 300.6 | 318.1 | 196.6 | 13.3 | 7.7 | 9.8 | 7.5 | 7.9 | 5.4 | 46.4 | 28.7 |
| | | 16 | MM | F | 2.0 | 399 | 73 | 27 | 190 | 189 | (Day 46) | -0.5 | 1246.4 | 585.0 | 670.4 | 448.0 | 522.5 | 149.7 | 17.3 | 8.5 | 10.8 | 7.2 | 9.3 | 3.5 | 41.9 | 12.0 |
| | | 17 | MM | M | 2.0 | 98 | 65 | 32 | 25 | 26 | (Day 43) | 4.0 | 1139.3 | 596.2 | 454.9 | 219.7 | 390.1 | 288.3 | 17.1 | 9.7 | 8.3 | 4.7 | 7.2 | 5.2 | 34.2 | 25.3 |
| | | 18 | MM | M | 2.0 | 230 | 72 | 24 | 90 | 90 | (Day 43) | 0.0 | 1281.9 | 499.3 | 557.8 | 329.5 | 482.9 | 233.4 | 30.8 | 15.2 | 16.6 | 10.3 | 14.6 | 8.2 | 37.6 | 18.2 |
| | PD | 19 | UTC | F | 2.0 | 47 | 69 | 23 | 153 | 238 | (Day 49) | 55.6 | 561.4 | 188.9 | 160.4 | N.A. | N.A. | N.A. | 7.8 | 3.2 | 2.8 | N.A. | N.A. | N.A. | N.A. | N.A. |
| | | 20 | MM | M | 4.0 | 35 | 73 | 25 | 119 | 129.4 | (Day 43) | 8.5 | 830.8 | 385.4 | 317.7 | 288.2 | 215.8 | 191.2 | 10.6 | 5.8 | 4.6 | 3.9 | 2.9 | 2.6 | 26.0 | 23.0 |
| | | 21 | MM | F | 6.0 | 33 | 69 | 30 | 230 | 234.4 | (Day 36) | 2.0 | 721.1 | 352.5 | 199.0 | 164.0 | 177.2 | 161.6 | 9.7 | 5.6 | 3.4 | 2.7 | 2.4 | 2.7 | 24.6 | 22.4 |
| | SD | 22 | MM | F | 2.0 | 41 | 59 | 28 | 213 | 226 | (Day 43) | 6.1 | 1348.4 | 758.3 | 250.7 | 275.7 | 227.3 | 234.8 | 16.6 | 9.5 | 3.2 | 3.7 | 2.5 | 2.7 | 16.8 | 17.4 |
| | | 23 | MM | F | 2.0 | 184 | 68 | 17 | 43 | 44 | (Day 36) | 2.3 | 759.2 | 230.9 | 149.3 | 72.0 | 113.3 | 82.3 | 10.4 | 4.0 | 2.7 | 1.7 | 2.3 | 1.9 | 14.9 | 10.8 |
| | | 24 | MM | M | 4.0 | 59 | 76 | 24 | 68 | 79 | (Day 42) | 16.2 | 998.5 | 526.7 | 237.8 | 252.4 | N.A. | N.A. | 11.2 | 6.4 | 2.5 | 2.6 | N.A. | N.A. | N.A. | N.A. |
| | | 25 | MM | F | 6.0 | 83 | 73 | 23 | 104 | 113 | (Day 42) | 8.7 | 712.1 | 349.8 | 121.9 | 110.2 | 140.6 | 144.2 | 11.1 | 4.8 | 2.2 | 2.4 | 3.0 | 2.5 | 19.7 | 20.2 |
| | | 26 | MM | F | 6.0 | 258 | 64 | 26 | 194 | 178.9 | (Day 43) | -7.5 | 1027.4 | 578.2 | 285.6 | 287.1 | 302.9 | 261.9 | 12.0 | 6.5 | 2.6 | 3.0 | 2.8 | 2.8 | 29.5 | 25.5 |
Baseline
(Day1Pre)
Once per two weeks
 (Q2W)
Once a week
 (Q1W)
Abbreviations: BMI=body mass index; MM=malignant mesothelioma; N.A.=not assessed; PD=progressive disease; PFS=progression free survival; RCC=renal cell carcinoma;
SD=stable disease; UTC=urothelial carcinoma;
