# Supplementary material for: Serum soluble CD26/DPP4 titer variation is a potential prognostic biomarker in cancer therapy with a humanized anti-CD26 antibody
Source: Biomark Res. 2021 Mar 23;9:21. doi: 10.1186/s40364-021-00273-0 (PMC7989014; doi:10.1186/s40364-021-00273-0)
Supplement: Supplementary file 2 — Additional file 2: Table S2. Detailed information about 26 evaluable cases [file 40364_2021_273_MOESM2_ESM.pptx]

## Slide 1
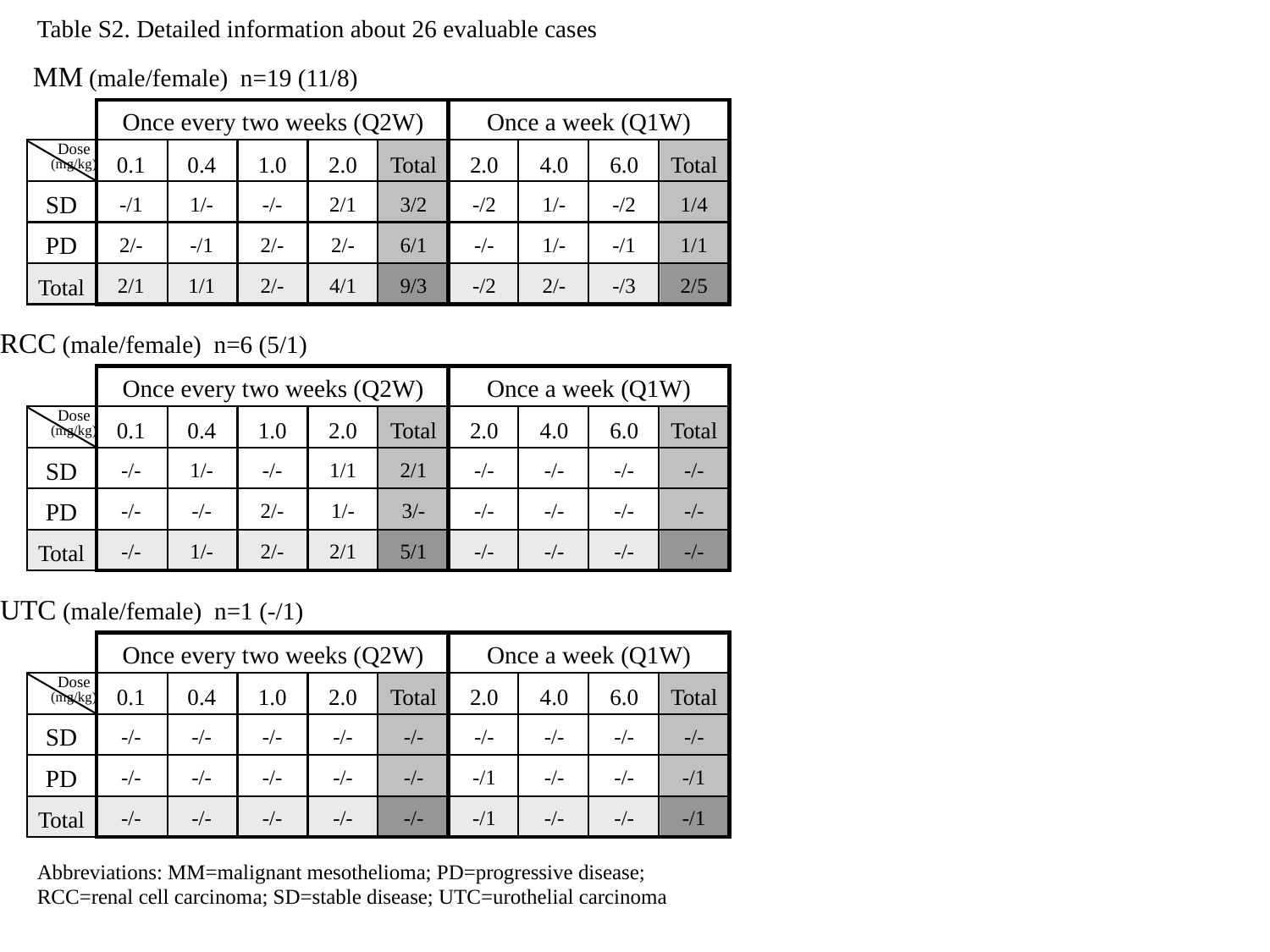

Table S2. Detailed information about 26 evaluable cases
MM (male/female) n=19 (11/8)
Once every two weeks (Q2W)
Once a week (Q1W)
Dose
(mg/kg)
0.1
0.4
1.0
2.0
Total
2.0
4.0
6.0
Total
SD
-/1
1/-
-/-
2/1
3/2
-/2
1/-
-/2
1/4
PD
2/-
-/1
2/-
2/-
6/1
-/-
1/-
-/1
1/1
2/1
1/1
2/-
4/1
9/3
-/2
2/-
-/3
2/5
Total
RCC (male/female) n=6 (5/1)
Once every two weeks (Q2W)
Once a week (Q1W)
Dose
(mg/kg)
0.1
0.4
1.0
2.0
Total
2.0
4.0
6.0
Total
SD
-/-
1/-
-/-
1/1
2/1
-/-
-/-
-/-
-/-
PD
-/-
-/-
2/-
1/-
3/-
-/-
-/-
-/-
-/-
-/-
1/-
2/-
2/1
5/1
-/-
-/-
-/-
-/-
Total
UTC (male/female) n=1 (-/1)
Once every two weeks (Q2W)
Once a week (Q1W)
Dose
(mg/kg)
0.1
0.4
1.0
2.0
Total
2.0
4.0
6.0
Total
SD
-/-
-/-
-/-
-/-
-/-
-/-
-/-
-/-
-/-
PD
-/-
-/-
-/-
-/-
-/-
-/1
-/-
-/-
-/1
-/-
-/-
-/-
-/-
-/-
-/1
-/-
-/-
-/1
Total
Abbreviations: MM=malignant mesothelioma; PD=progressive disease;
RCC=renal cell carcinoma; SD=stable disease; UTC=urothelial carcinoma
