# Supplementary material for: Serum soluble CD26/DPP4 titer variation is a potential prognostic biomarker in cancer therapy with a humanized anti-CD26 antibody
Source: Biomark Res. 2021 Mar 23;9:21. doi: 10.1186/s40364-021-00273-0 (PMC7989014; doi:10.1186/s40364-021-00273-0)
Supplement: Supplementary file 3 — Additional file 3: Figure S1. Correlation between serum soluble CD26 level and DPP4 enzyme activity [file 40364_2021_273_MOESM3_ESM.pptx]

## Slide 1
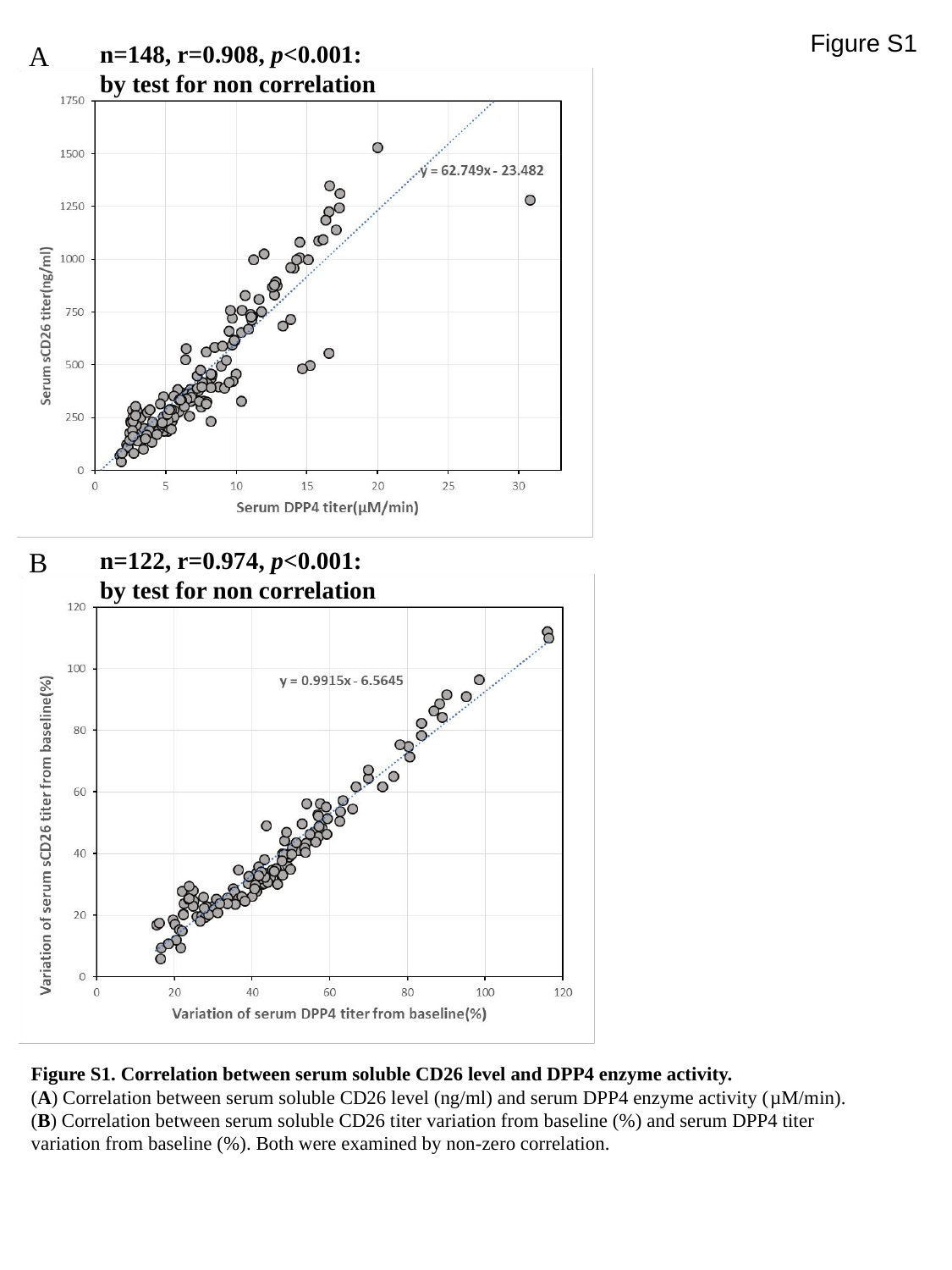

Figure S1
A
n=148, r=0.908, p<0.001:
by test for non correlation
B
n=122, r=0.974, p<0.001:
by test for non correlation
Figure S1. Correlation between serum soluble CD26 level and DPP4 enzyme activity.
(A) Correlation between serum soluble CD26 level (ng/ml) and serum DPP4 enzyme activity (µM/min).
(B) Correlation between serum soluble CD26 titer variation from baseline (%) and serum DPP4 titer
variation from baseline (%). Both were examined by non-zero correlation.
