# Supplementary material for: Serum soluble CD26/DPP4 titer variation is a potential prognostic biomarker in cancer therapy with a humanized anti-CD26 antibody
Source: Biomark Res. 2021 Mar 23;9:21. doi: 10.1186/s40364-021-00273-0 (PMC7989014; doi:10.1186/s40364-021-00273-0)
Supplement: Supplementary file 4 — Additional file 4: Table S3. Correlation between serum sCD26/DPP4 titer variation (%) and tumor volume change (%) or PFS (days) in 18 cases with Q2W administration by PPMC or SRDC analysis [file 40364_2021_273_MOESM4_ESM.pptx]

## Slide 1
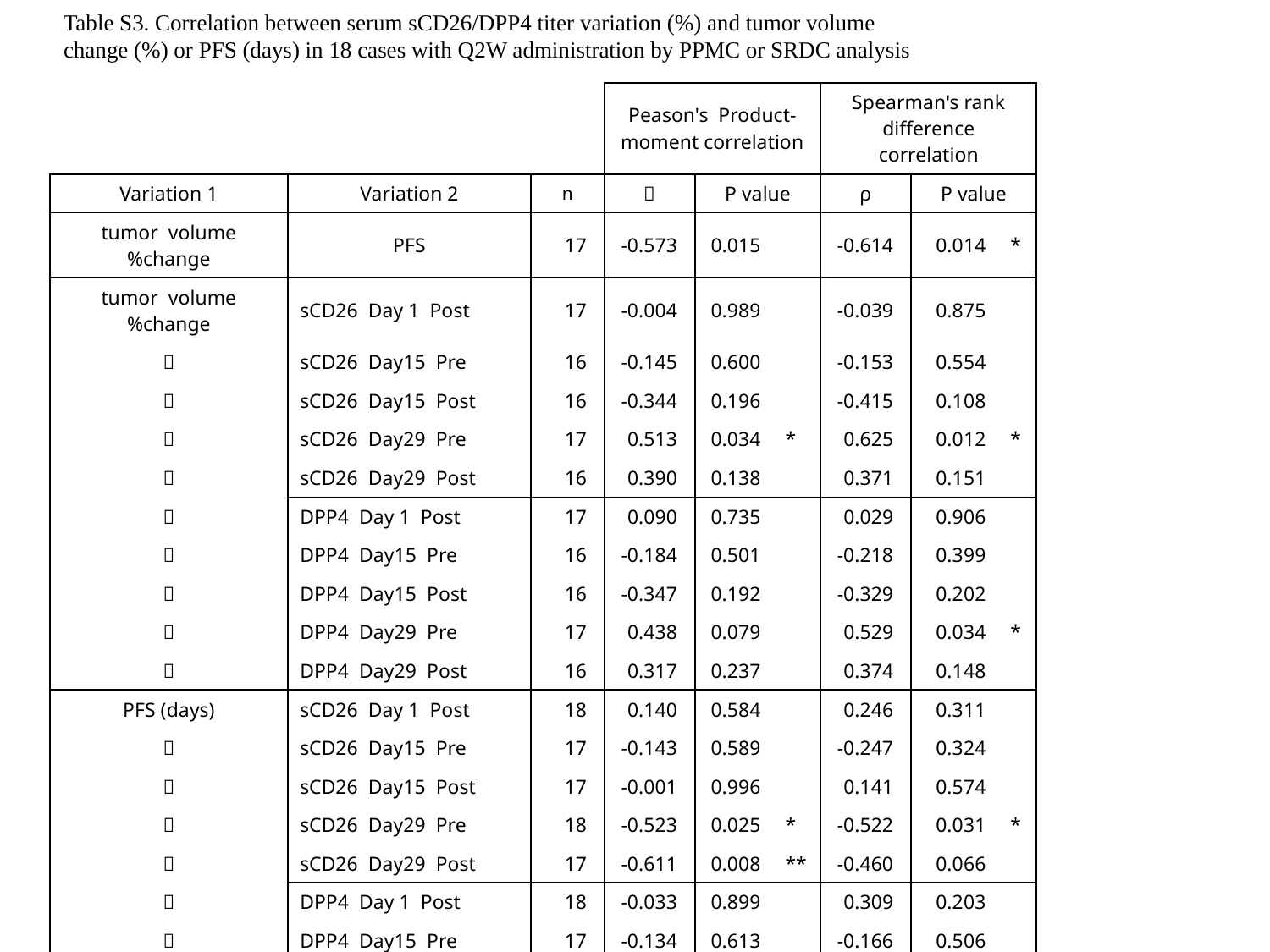

Table S3. Correlation between serum sCD26/DPP4 titer variation (%) and tumor volume
change (%) or PFS (days) in 18 cases with Q2W administration by PPMC or SRDC analysis
| | | | Peason's Product-moment correlation | | | Spearman's rank difference correlation | | |
| --- | --- | --- | --- | --- | --- | --- | --- | --- |
| Variation 1 | Variation 2 | n | ｒ | P value | | ρ | P value | |
| tumor volume %change | PFS | 17 | -0.573 | 0.015 | | -0.614 | 0.014 | \* |
| tumor volume %change | sCD26 Day 1 Post | 17 | -0.004 | 0.989 | | -0.039 | 0.875 | |
| 〃 | sCD26 Day15 Pre | 16 | -0.145 | 0.600 | | -0.153 | 0.554 | |
| 〃 | sCD26 Day15 Post | 16 | -0.344 | 0.196 | | -0.415 | 0.108 | |
| 〃 | sCD26 Day29 Pre | 17 | 0.513 | 0.034 | \* | 0.625 | 0.012 | \* |
| 〃 | sCD26 Day29 Post | 16 | 0.390 | 0.138 | | 0.371 | 0.151 | |
| 〃 | DPP4 Day 1 Post | 17 | 0.090 | 0.735 | | 0.029 | 0.906 | |
| 〃 | DPP4 Day15 Pre | 16 | -0.184 | 0.501 | | -0.218 | 0.399 | |
| 〃 | DPP4 Day15 Post | 16 | -0.347 | 0.192 | | -0.329 | 0.202 | |
| 〃 | DPP4 Day29 Pre | 17 | 0.438 | 0.079 | | 0.529 | 0.034 | \* |
| 〃 | DPP4 Day29 Post | 16 | 0.317 | 0.237 | | 0.374 | 0.148 | |
| PFS (days) | sCD26 Day 1 Post | 18 | 0.140 | 0.584 | | 0.246 | 0.311 | |
| 〃 | sCD26 Day15 Pre | 17 | -0.143 | 0.589 | | -0.247 | 0.324 | |
| 〃 | sCD26 Day15 Post | 17 | -0.001 | 0.996 | | 0.141 | 0.574 | |
| 〃 | sCD26 Day29 Pre | 18 | -0.523 | 0.025 | \* | -0.522 | 0.031 | \* |
| 〃 | sCD26 Day29 Post | 17 | -0.611 | 0.008 | \*\* | -0.460 | 0.066 | |
| 〃 | DPP4 Day 1 Post | 18 | -0.033 | 0.899 | | 0.309 | 0.203 | |
| 〃 | DPP4 Day15 Pre | 17 | -0.134 | 0.613 | | -0.166 | 0.506 | |
| 〃 | DPP4 Day15 Post | 17 | -0.043 | 0.871 | | -0.020 | 0.937 | |
| 〃 | DPP4 Day29 Pre | 18 | -0.425 | 0.079 | | -0.418 | 0.085 | |
| 〃 | DPP4 Day29 Post | 17 | -0.580 | 0.013 | \* | -0.515 | 0.039 | \* |
