# Supplementary material for: Serum soluble CD26/DPP4 titer variation is a potential prognostic biomarker in cancer therapy with a humanized anti-CD26 antibody
Source: Biomark Res. 2021 Mar 23;9:21. doi: 10.1186/s40364-021-00273-0 (PMC7989014; doi:10.1186/s40364-021-00273-0)
Supplement: Supplementary file 5 — Additional file 5: Table S4. Correlation between serum sCD26/DPP4 titer variation (%) and tumor volume change (%) or PFS (days) in 14 male cases with Q2W administration by PPMC or SRDC analysis [file 40364_2021_273_MOESM5_ESM.pptx]

## Slide 1
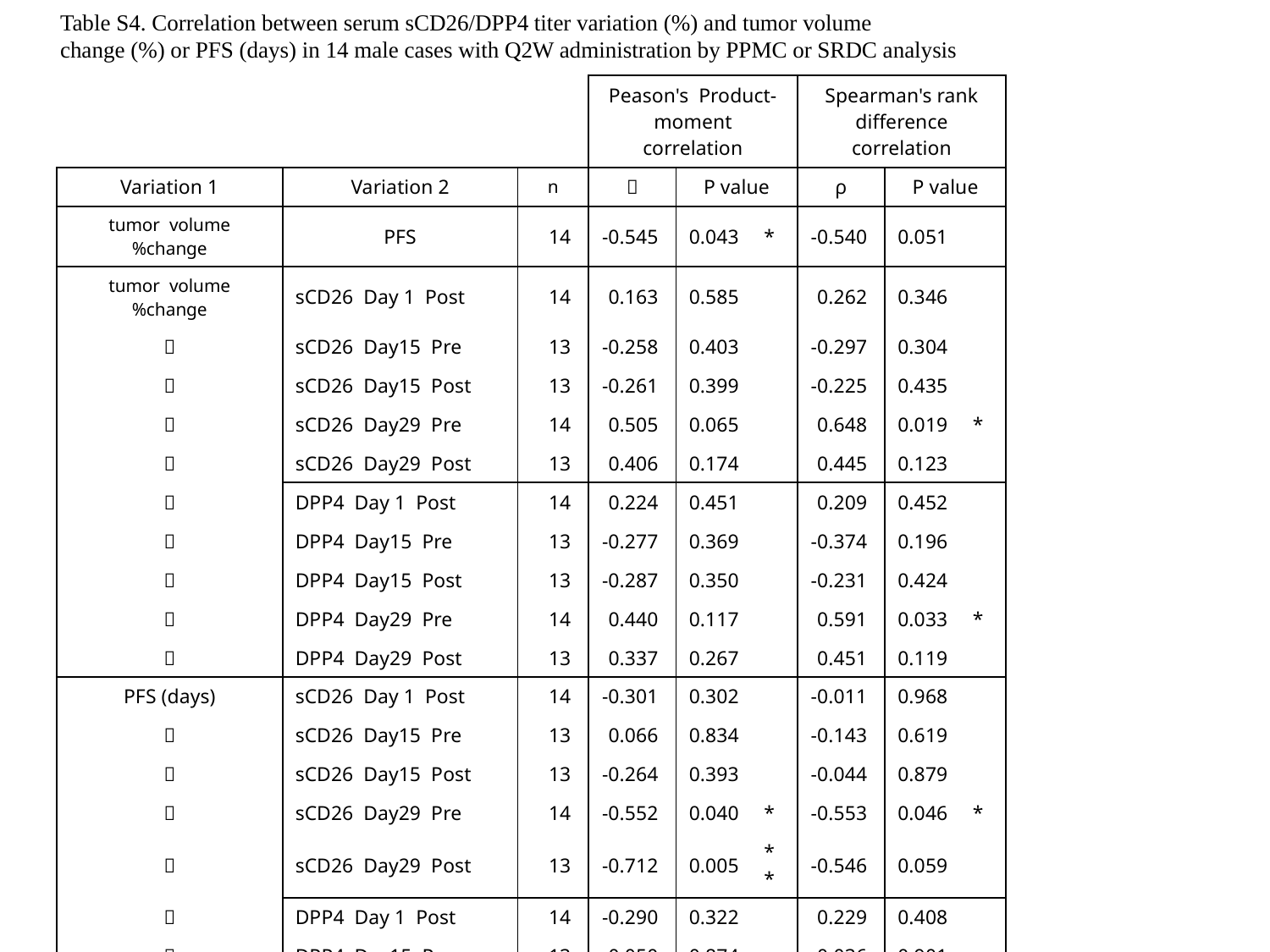

Table S4. Correlation between serum sCD26/DPP4 titer variation (%) and tumor volume
change (%) or PFS (days) in 14 male cases with Q2W administration by PPMC or SRDC analysis
| | | | Peason's Product-moment correlation | | | Spearman's rank difference correlation | | |
| --- | --- | --- | --- | --- | --- | --- | --- | --- |
| Variation 1 | Variation 2 | n | ｒ | P value | | ρ | P value | |
| tumor volume %change | PFS | 14 | -0.545 | 0.043 | \* | -0.540 | 0.051 | |
| tumor volume %change | sCD26 Day 1 Post | 14 | 0.163 | 0.585 | | 0.262 | 0.346 | |
| 〃 | sCD26 Day15 Pre | 13 | -0.258 | 0.403 | | -0.297 | 0.304 | |
| 〃 | sCD26 Day15 Post | 13 | -0.261 | 0.399 | | -0.225 | 0.435 | |
| 〃 | sCD26 Day29 Pre | 14 | 0.505 | 0.065 | | 0.648 | 0.019 | \* |
| 〃 | sCD26 Day29 Post | 13 | 0.406 | 0.174 | | 0.445 | 0.123 | |
| 〃 | DPP4 Day 1 Post | 14 | 0.224 | 0.451 | | 0.209 | 0.452 | |
| 〃 | DPP4 Day15 Pre | 13 | -0.277 | 0.369 | | -0.374 | 0.196 | |
| 〃 | DPP4 Day15 Post | 13 | -0.287 | 0.350 | | -0.231 | 0.424 | |
| 〃 | DPP4 Day29 Pre | 14 | 0.440 | 0.117 | | 0.591 | 0.033 | \* |
| 〃 | DPP4 Day29 Post | 13 | 0.337 | 0.267 | | 0.451 | 0.119 | |
| PFS (days) | sCD26 Day 1 Post | 14 | -0.301 | 0.302 | | -0.011 | 0.968 | |
| 〃 | sCD26 Day15 Pre | 13 | 0.066 | 0.834 | | -0.143 | 0.619 | |
| 〃 | sCD26 Day15 Post | 13 | -0.264 | 0.393 | | -0.044 | 0.879 | |
| 〃 | sCD26 Day29 Pre | 14 | -0.552 | 0.040 | \* | -0.553 | 0.046 | \* |
| 〃 | sCD26 Day29 Post | 13 | -0.712 | 0.005 | \*\* | -0.546 | 0.059 | |
| 〃 | DPP4 Day 1 Post | 14 | -0.290 | 0.322 | | 0.229 | 0.408 | |
| 〃 | DPP4 Day15 Pre | 13 | 0.050 | 0.874 | | -0.036 | 0.901 | |
| 〃 | DPP4 Day15 Post | 13 | -0.211 | 0.499 | | -0.108 | 0.709 | |
| 〃 | DPP4 Day29 Pre | 14 | -0.451 | 0.107 | | -0.474 | 0.087 | |
| 〃 | DPP4 Day29 Post | 13 | -0.672 | 0.010 | \* | -0.654 | 0.024 | \* |
