# Supplementary material for: Serum soluble CD26/DPP4 titer variation is a potential prognostic biomarker in cancer therapy with a humanized anti-CD26 antibody
Source: Biomark Res. 2021 Mar 23;9:21. doi: 10.1186/s40364-021-00273-0 (PMC7989014; doi:10.1186/s40364-021-00273-0)
Supplement: Supplementary file 6 — Additional file 6: Table S5. Correlation between serum sCD26/DPP4 titer variation (%) and tumor volume change (%) or PFS (days) in 19 MM cases by PPMC or SRDC analysis. [file 40364_2021_273_MOESM6_ESM.pptx]

## Slide 1
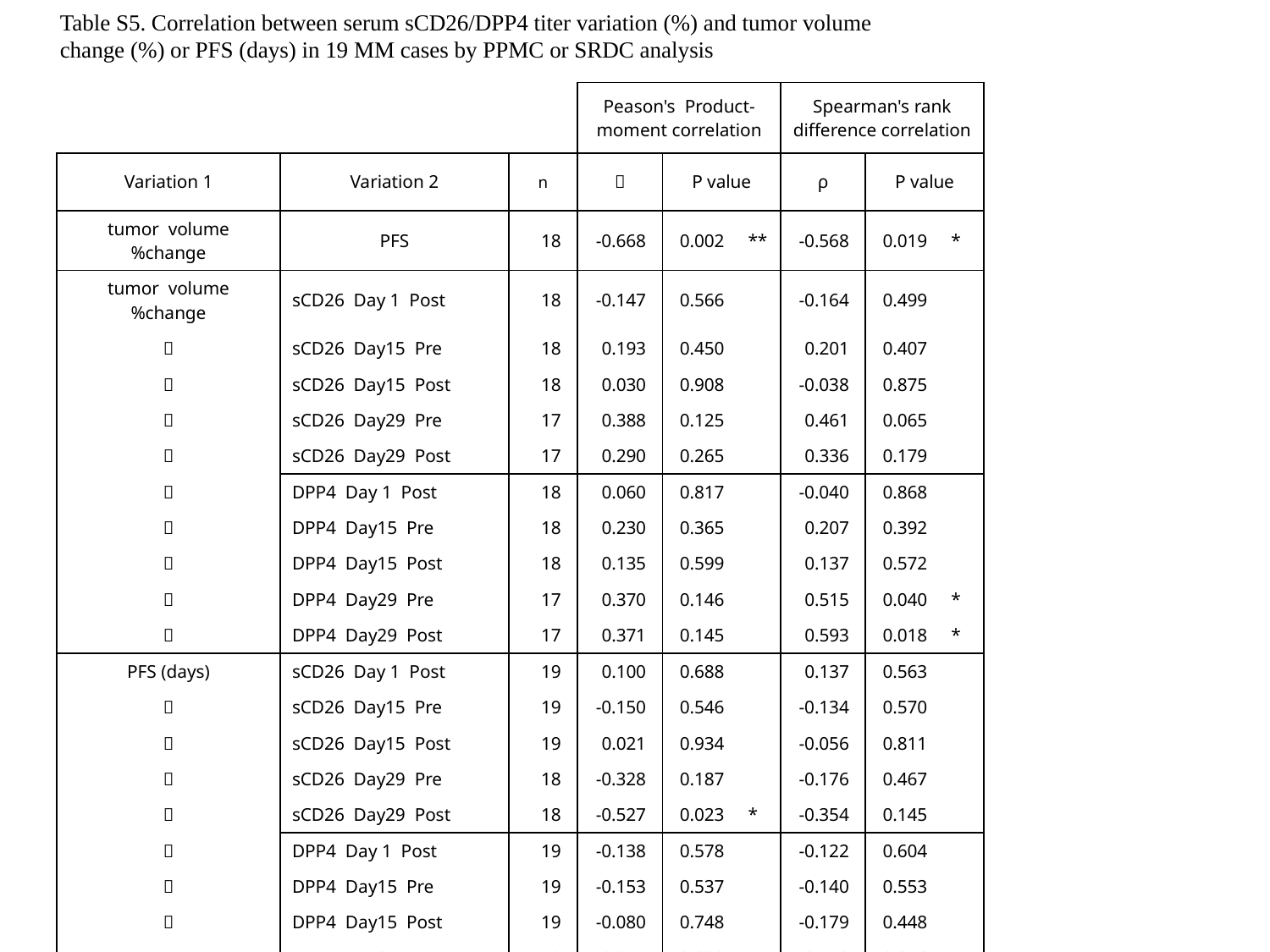

Table S5. Correlation between serum sCD26/DPP4 titer variation (%) and tumor volume
change (%) or PFS (days) in 19 MM cases by PPMC or SRDC analysis
| | | | Peason's Product-moment correlation | | | Spearman's rank difference correlation | | |
| --- | --- | --- | --- | --- | --- | --- | --- | --- |
| Variation 1 | Variation 2 | n | ｒ | P value | | ρ | P value | |
| tumor volume %change | PFS | 18 | -0.668 | 0.002 | \*\* | -0.568 | 0.019 | \* |
| tumor volume %change | sCD26 Day 1 Post | 18 | -0.147 | 0.566 | | -0.164 | 0.499 | |
| 〃 | sCD26 Day15 Pre | 18 | 0.193 | 0.450 | | 0.201 | 0.407 | |
| 〃 | sCD26 Day15 Post | 18 | 0.030 | 0.908 | | -0.038 | 0.875 | |
| 〃 | sCD26 Day29 Pre | 17 | 0.388 | 0.125 | | 0.461 | 0.065 | |
| 〃 | sCD26 Day29 Post | 17 | 0.290 | 0.265 | | 0.336 | 0.179 | |
| 〃 | DPP4 Day 1 Post | 18 | 0.060 | 0.817 | | -0.040 | 0.868 | |
| 〃 | DPP4 Day15 Pre | 18 | 0.230 | 0.365 | | 0.207 | 0.392 | |
| 〃 | DPP4 Day15 Post | 18 | 0.135 | 0.599 | | 0.137 | 0.572 | |
| 〃 | DPP4 Day29 Pre | 17 | 0.370 | 0.146 | | 0.515 | 0.040 | \* |
| 〃 | DPP4 Day29 Post | 17 | 0.371 | 0.145 | | 0.593 | 0.018 | \* |
| PFS (days) | sCD26 Day 1 Post | 19 | 0.100 | 0.688 | | 0.137 | 0.563 | |
| 〃 | sCD26 Day15 Pre | 19 | -0.150 | 0.546 | | -0.134 | 0.570 | |
| 〃 | sCD26 Day15 Post | 19 | 0.021 | 0.934 | | -0.056 | 0.811 | |
| 〃 | sCD26 Day29 Pre | 18 | -0.328 | 0.187 | | -0.176 | 0.467 | |
| 〃 | sCD26 Day29 Post | 18 | -0.527 | 0.023 | \* | -0.354 | 0.145 | |
| 〃 | DPP4 Day 1 Post | 19 | -0.138 | 0.578 | | -0.122 | 0.604 | |
| 〃 | DPP4 Day15 Pre | 19 | -0.153 | 0.537 | | -0.140 | 0.553 | |
| 〃 | DPP4 Day15 Post | 19 | -0.080 | 0.748 | | -0.179 | 0.448 | |
| 〃 | DPP4 Day29 Pre | 18 | -0.251 | 0.320 | | -0.210 | 0.386 | |
| 〃 | DPP4 Day29 Post | 18 | -0.457 | 0.056 | | -0.441 | 0.069 | |
