# Supplementary material for: Serum soluble CD26/DPP4 titer variation is a potential prognostic biomarker in cancer therapy with a humanized anti-CD26 antibody
Source: Biomark Res. 2021 Mar 23;9:21. doi: 10.1186/s40364-021-00273-0 (PMC7989014; doi:10.1186/s40364-021-00273-0)
Supplement: Supplementary file 7 — Additional file 7: Table S6. Correlation between serum sCD26/DPP4 titer variation (%) and tumor volume change (%) or PFS (days) in 12 MM cases with Q2W administration by PPMC or SRDC analysis. [file 40364_2021_273_MOESM7_ESM.pptx]

## Slide 1
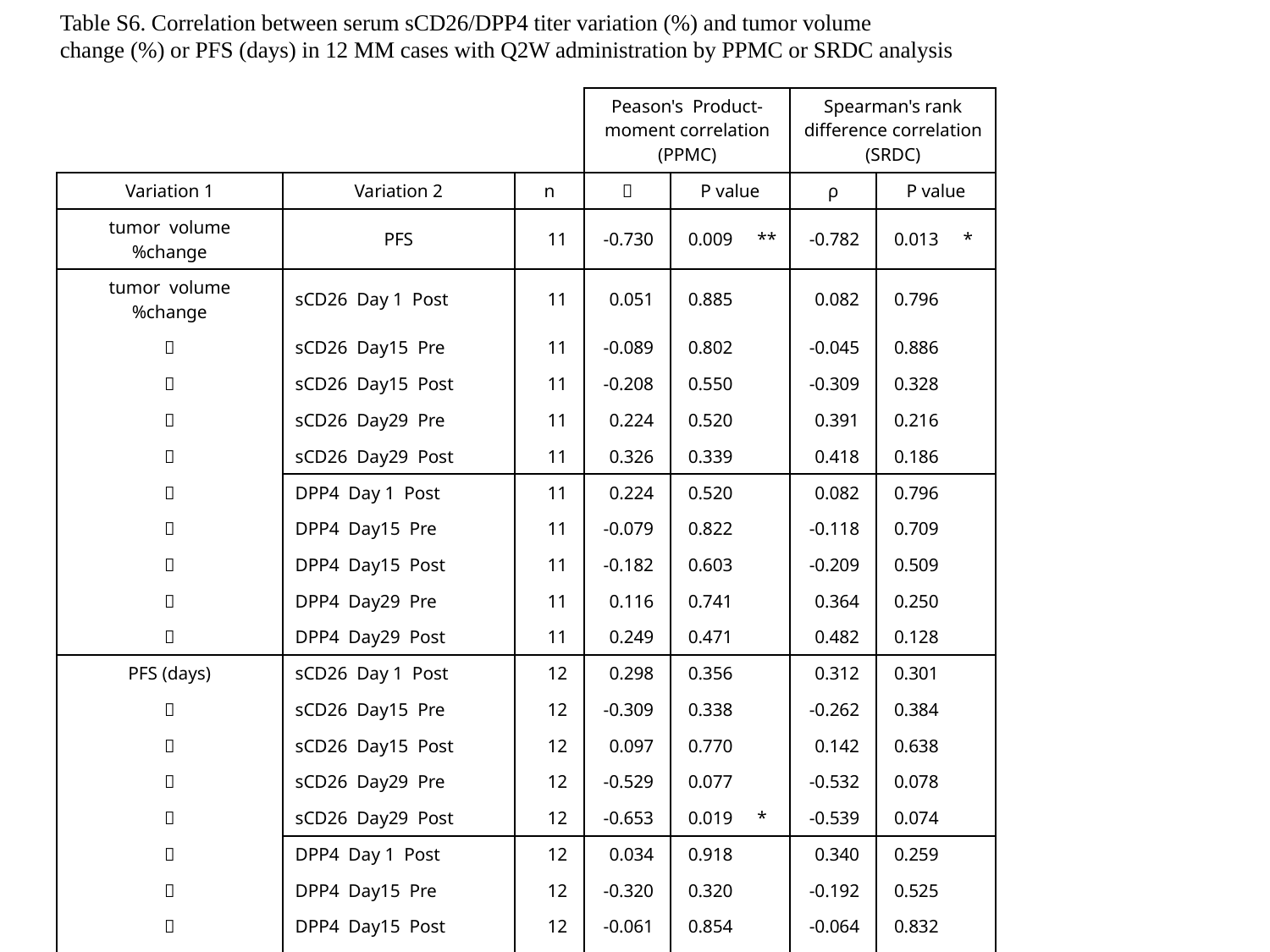

Table S6. Correlation between serum sCD26/DPP4 titer variation (%) and tumor volume
change (%) or PFS (days) in 12 MM cases with Q2W administration by PPMC or SRDC analysis
| | | | Peason's Product-moment correlation (PPMC) | | | Spearman's rank difference correlation (SRDC) | | |
| --- | --- | --- | --- | --- | --- | --- | --- | --- |
| Variation 1 | Variation 2 | n | ｒ | P value | | ρ | P value | |
| tumor volume %change | PFS | 11 | -0.730 | 0.009 | \*\* | -0.782 | 0.013 | \* |
| tumor volume %change | sCD26 Day 1 Post | 11 | 0.051 | 0.885 | | 0.082 | 0.796 | |
| 〃 | sCD26 Day15 Pre | 11 | -0.089 | 0.802 | | -0.045 | 0.886 | |
| 〃 | sCD26 Day15 Post | 11 | -0.208 | 0.550 | | -0.309 | 0.328 | |
| 〃 | sCD26 Day29 Pre | 11 | 0.224 | 0.520 | | 0.391 | 0.216 | |
| 〃 | sCD26 Day29 Post | 11 | 0.326 | 0.339 | | 0.418 | 0.186 | |
| 〃 | DPP4 Day 1 Post | 11 | 0.224 | 0.520 | | 0.082 | 0.796 | |
| 〃 | DPP4 Day15 Pre | 11 | -0.079 | 0.822 | | -0.118 | 0.709 | |
| 〃 | DPP4 Day15 Post | 11 | -0.182 | 0.603 | | -0.209 | 0.509 | |
| 〃 | DPP4 Day29 Pre | 11 | 0.116 | 0.741 | | 0.364 | 0.250 | |
| 〃 | DPP4 Day29 Post | 11 | 0.249 | 0.471 | | 0.482 | 0.128 | |
| PFS (days) | sCD26 Day 1 Post | 12 | 0.298 | 0.356 | | 0.312 | 0.301 | |
| 〃 | sCD26 Day15 Pre | 12 | -0.309 | 0.338 | | -0.262 | 0.384 | |
| 〃 | sCD26 Day15 Post | 12 | 0.097 | 0.770 | | 0.142 | 0.638 | |
| 〃 | sCD26 Day29 Pre | 12 | -0.529 | 0.077 | | -0.532 | 0.078 | |
| 〃 | sCD26 Day29 Post | 12 | -0.653 | 0.019 | \* | -0.539 | 0.074 | |
| 〃 | DPP4 Day 1 Post | 12 | 0.034 | 0.918 | | 0.340 | 0.259 | |
| 〃 | DPP4 Day15 Pre | 12 | -0.320 | 0.320 | | -0.192 | 0.525 | |
| 〃 | DPP4 Day15 Post | 12 | -0.061 | 0.854 | | -0.064 | 0.832 | |
| 〃 | DPP4 Day29 Pre | 12 | -0.446 | 0.150 | | -0.525 | 0.082 | |
| 〃 | DPP4 Day29 Post | 12 | -0.629 | 0.026 | \* | -0.624 | 0.038 | \* |
