# Supplementary material for: Serum soluble CD26/DPP4 titer variation is a potential prognostic biomarker in cancer therapy with a humanized anti-CD26 antibody
Source: Biomark Res. 2021 Mar 23;9:21. doi: 10.1186/s40364-021-00273-0 (PMC7989014; doi:10.1186/s40364-021-00273-0)
Supplement: Supplementary file 8 — Additional file 8: Table S7. Correlation between serum sCD26/DPP4 titer variation (%) and tumor volume change (%) or PFS (days) in 9 MM, male cases with Q2W administration by PPMC or SRDC analysis. [file 40364_2021_273_MOESM8_ESM.pptx]

## Slide 1
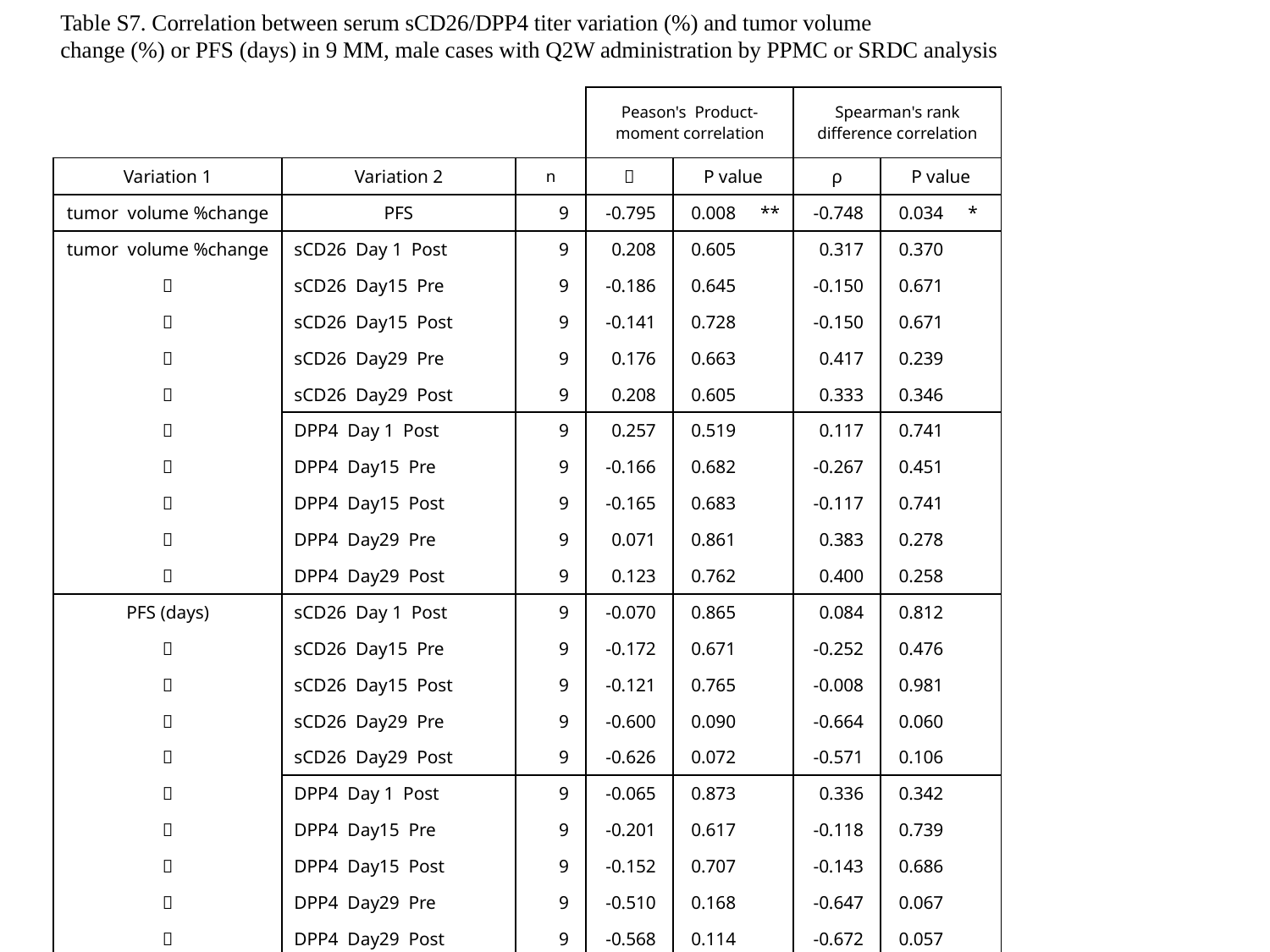

Table S7. Correlation between serum sCD26/DPP4 titer variation (%) and tumor volume
change (%) or PFS (days) in 9 MM, male cases with Q2W administration by PPMC or SRDC analysis
| | | | Peason's Product-moment correlation | | | Spearman's rank difference correlation | | |
| --- | --- | --- | --- | --- | --- | --- | --- | --- |
| Variation 1 | Variation 2 | n | ｒ | P value | | ρ | P value | |
| tumor volume %change | PFS | 9 | -0.795 | 0.008 | \*\* | -0.748 | 0.034 | \* |
| tumor volume %change | sCD26 Day 1 Post | 9 | 0.208 | 0.605 | | 0.317 | 0.370 | |
| 〃 | sCD26 Day15 Pre | 9 | -0.186 | 0.645 | | -0.150 | 0.671 | |
| 〃 | sCD26 Day15 Post | 9 | -0.141 | 0.728 | | -0.150 | 0.671 | |
| 〃 | sCD26 Day29 Pre | 9 | 0.176 | 0.663 | | 0.417 | 0.239 | |
| 〃 | sCD26 Day29 Post | 9 | 0.208 | 0.605 | | 0.333 | 0.346 | |
| 〃 | DPP4 Day 1 Post | 9 | 0.257 | 0.519 | | 0.117 | 0.741 | |
| 〃 | DPP4 Day15 Pre | 9 | -0.166 | 0.682 | | -0.267 | 0.451 | |
| 〃 | DPP4 Day15 Post | 9 | -0.165 | 0.683 | | -0.117 | 0.741 | |
| 〃 | DPP4 Day29 Pre | 9 | 0.071 | 0.861 | | 0.383 | 0.278 | |
| 〃 | DPP4 Day29 Post | 9 | 0.123 | 0.762 | | 0.400 | 0.258 | |
| PFS (days) | sCD26 Day 1 Post | 9 | -0.070 | 0.865 | | 0.084 | 0.812 | |
| 〃 | sCD26 Day15 Pre | 9 | -0.172 | 0.671 | | -0.252 | 0.476 | |
| 〃 | sCD26 Day15 Post | 9 | -0.121 | 0.765 | | -0.008 | 0.981 | |
| 〃 | sCD26 Day29 Pre | 9 | -0.600 | 0.090 | | -0.664 | 0.060 | |
| 〃 | sCD26 Day29 Post | 9 | -0.626 | 0.072 | | -0.571 | 0.106 | |
| 〃 | DPP4 Day 1 Post | 9 | -0.065 | 0.873 | | 0.336 | 0.342 | |
| 〃 | DPP4 Day15 Pre | 9 | -0.201 | 0.617 | | -0.118 | 0.739 | |
| 〃 | DPP4 Day15 Post | 9 | -0.152 | 0.707 | | -0.143 | 0.686 | |
| 〃 | DPP4 Day29 Pre | 9 | -0.510 | 0.168 | | -0.647 | 0.067 | |
| 〃 | DPP4 Day29 Post | 9 | -0.568 | 0.114 | | -0.672 | 0.057 | |
