# Supplementary material for: Serum soluble CD26/DPP4 titer variation is a potential prognostic biomarker in cancer therapy with a humanized anti-CD26 antibody
Source: Biomark Res. 2021 Mar 23;9:21. doi: 10.1186/s40364-021-00273-0 (PMC7989014; doi:10.1186/s40364-021-00273-0)
Supplement: Supplementary file 9 — Additional file 9: Figure S2. Cell surface protein expression of CD26 on the human tumor and non-tumor cells. [file 40364_2021_273_MOESM9_ESM.pptx]

## Slide 1
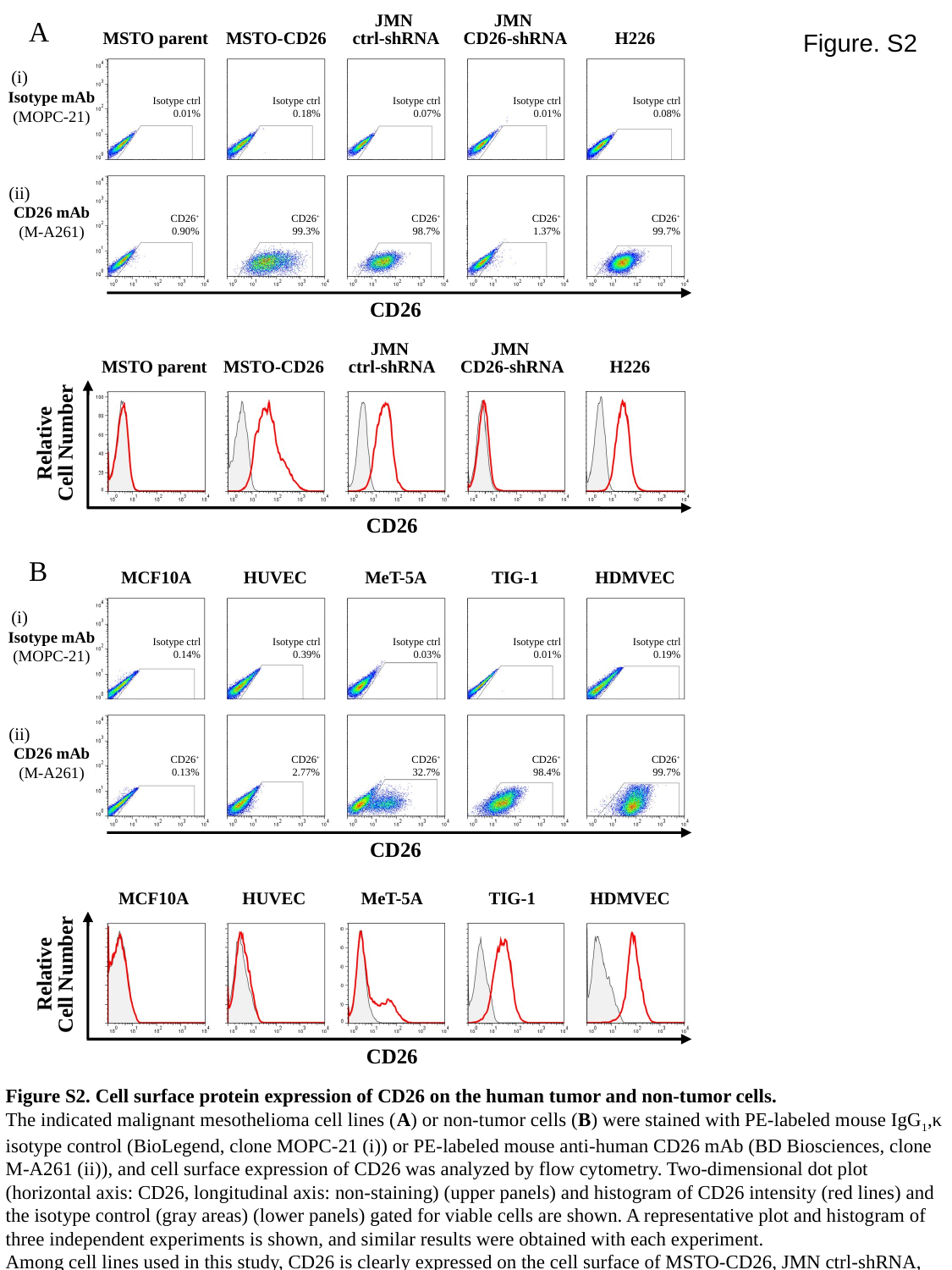

JMN
ctrl-shRNA
JMN
CD26-shRNA
A
MSTO parent
MSTO-CD26
H226
Figure. S2
(i)
Isotype ctrl
0.01%
Isotype ctrl
0.18%
Isotype ctrl
0.07%
Isotype ctrl
0.01%
Isotype ctrl
0.08%
Isotype mAb
(MOPC-21)
(ii)
CD26+
0.90%
CD26+
99.3%
CD26+
98.7%
CD26+
1.37%
CD26+
99.7%
CD26 mAb
(M-A261)
CD26
JMN
ctrl-shRNA
JMN
CD26-shRNA
MSTO parent
MSTO-CD26
H226
Relative
Cell Number
CD26
B
MCF10A
HUVEC
HDMVEC
MeT-5A
TIG-1
(i)
Isotype ctrl
0.14%
Isotype ctrl
0.39%
Isotype ctrl
0.03%
Isotype ctrl
0.01%
Isotype ctrl
0.19%
Isotype mAb
(MOPC-21)
(ii)
CD26+
0.13%
CD26+
2.77%
CD26+
32.7%
CD26+
98.4%
CD26+
99.7%
CD26 mAb
(M-A261)
CD26
MCF10A
HUVEC
HDMVEC
MeT-5A
TIG-1
Relative
Cell Number
CD26
Figure S2. Cell surface protein expression of CD26 on the human tumor and non-tumor cells.
The indicated malignant mesothelioma cell lines (A) or non-tumor cells (B) were stained with PE-labeled mouse IgG1,κ
isotype control (BioLegend, clone MOPC-21 (i)) or PE-labeled mouse anti-human CD26 mAb (BD Biosciences, clone
M-A261 (ii)), and cell surface expression of CD26 was analyzed by flow cytometry. Two-dimensional dot plot
(horizontal axis: CD26, longitudinal axis: non-staining) (upper panels) and histogram of CD26 intensity (red lines) and
the isotype control (gray areas) (lower panels) gated for viable cells are shown. A representative plot and histogram of
three independent experiments is shown, and similar results were obtained with each experiment.
Among cell lines used in this study, CD26 is clearly expressed on the cell surface of MSTO-CD26, JMN ctrl-shRNA,
H226, TIG-1 and HDMVEC, whereas CD26 is hardly expressed on MSTO parent, JMN CD26-shRNA, MCF10A and
HUVEC, and partially expressed on MeT-5A.
